# ACSL1‐Dependent Microglial Lipoimmunometabolic Reprogramming Underlies Cognitive Deficits in Alcohol Use Disorder

**DOI:** 10.1002/advs.202519760

**Published:** 2026-02-05

**Authors:** Liang Hao, Xing‐Rui Cao, Bai‐Qiang Li, Fu‐Ying Zhao, Jia‐Mei Wang, Rui‐Kang Gao, Zhi‐Peng Cao, Zhen‐Xian Du, Hua‐Qin Wang

**Affiliations:** ^1^ Department of Chemistry School of Forensic Medicine China Medical University Shenyang China; ^2^ Department of Biochemistry & Molecular Biology China Medical University Shenyang China; ^3^ Key Laboratory of Cell Biology, and Key Laboratory of Medical Cell Biology Ministry of Public Health Ministry of Education China Medical University Shenyang China; ^4^ National Clinical Research Center for Laboratory Medicine Department of Laboratory Medicine The First Hospital of China Medical University Shenyang China; ^5^ Department of Endocrinology & Metabolism The First Hospital of China Medical University Shenyang China; ^6^ Department of Forensic Pathology School of Forensic Medicine China Medical University Shenyang China; ^7^ Liaoning Province Key Laboratory of Forensic Bio‐Evidence Sciences Shenyang China

**Keywords:** ACSL1, alcohol use disorder (AUD), cognitive deficits, lipoimmunometabolic reprogramming, microglia

## Abstract

Alcohol use disorder (AUD) leads to cognitive impairment dependent on prefrontal cortex (PFC) dysfunction, yet the underlying cellular and molecular mechanisms, particularly the role of microglia, remain poorly understood. Through re‐analysis of single‐cell RNA sequencing data from AUD patients, we identified aberrant activation of lipid metabolic pathways in microglia and pinpointed acyl‐CoA synthetase long‐chain family member 1 (ACSL1) as a central regulator. In animal and cellular models, chronic ethanol exposure induced ACSL1 upregulation, triggering lipid droplet accumulation, neuroinflammatory activation, and aberrant microglia–neuron interactions mediated via PTPRM signaling. Pharmacological inhibition of ACSL1 reversed these pathological phenotypes. We further developed a dual‐targeted lipid nanoparticle system for microglia‐specific ACSL1 silencing, which effectively ameliorated ethanol‐induced cognitive deficits in mice. Our study unveils ACSL1‐mediated lipoimmunity reprogramming of microglia as a core mechanism underlying cognitive impairment in AUD and proposes a novel targeted therapeutic strategy.

## Graphical abstract

This study reveals that chronic alcohol exposure selectively upregulates ACSL1 expression in prefrontal cortical microglia, driving lipid metabolism reprogramming and lipid droplet accumulation, which activates the NLRP3 inflammasome and sustains neuroinflammation. To reverse this process, a dual‐targeted lipid nanoparticle, siACSL1@LNP‐MR, is designed to efficiently cross the blood‐brain barrier and specifically silence ACSL1 in microglia, significantly alleviating neuroinflammation and cognitive deficits.

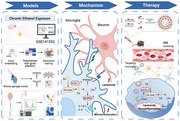

## 1 Introduction

Alcohol use disorder (AUD) represents a profound global health burden, characterized by compulsive alcohol use and a high propensity for relapse, contributing to millions of deaths annually and incurring substantial socioeconomic costs [1]. Beyond its well‐characterized effects on peripheral organs, chronic alcohol consumption inflicts profound damage on the central nervous system, leading to persistent cognitive deficits that severely impair quality of life and hinder recovery. Individuals with AUD commonly exhibit impairments in executive function, decision‐making, and memory [2, 3, 4]. These cognitive dysfunctions are not merely sequelae of intoxication but reflect enduring maladaptations in brain circuitry, particularly within the prefrontal cortex (PFC) [5, 6, 7, 8, 9].

Converging clinical evidence underscores the PFC as a critical locus of alcohol‐induced neuropathology [10, 11]. Neuroimaging studies in human patients with AUD consistently reveal structural and functional abnormalities in this region [12, 13]. For instance, positron emission tomography (PET) studies demonstrate significant hypometabolism in the medial and lateral PFC of abstinent patients with alcohol‐related cognitive impairment [14]. Similarly, meta‐analyses of functional magnetic resonance imaging (fMRI) data confirm that AUD is associated with aberrant activation patterns in the PFC, including the superior and middle frontal gyri, during tasks demanding inhibitory control, a core component of executive function [2]. This PFC dysfunction is hypothesized to underpin the loss of behavioral control and compulsive drinking patterns that characterize addiction [5, 6]. The vulnerability of the PFC is further highlighted in adolescence, a period of heightened neurodevelopment during which alcohol exposure can disrupt the maturation of corticolimbic circuits, thereby increasing the lifetime risk of developing AUD [15]. While the link between AUD, PFC dysfunction, and cognitive decline is well‐established, the precise cellular and molecular mechanisms within the PFC that drive this pathology remain incompletely understood [7, 10, 16].

As the resident immune sentinels of the central nervous system (CNS), microglia are pivotal in maintaining brain homeostasis through their roles in synaptic pruning, phagocytosis of cellular debris, and constant surveillance of the parenchyma [17, 18]. However, in response to pathological insults such as chronic alcohol exposure, microglia can undergo profound phenotypical remodeling, transitioning from a homeostatic state to a reactive one [10, 19]. This activation is a double‐edged sword: while essential for initial defense and repair, sustained or dysregulated microglial reactivity is a cornerstone of maladaptive neuroinflammation, contributing to synaptic dysfunction and neuronal damage, hallmarks of numerous neurodegenerative and neuropsychiatric disorders, including AUD [20, 21, 22].

In recent years, the emerging field of immunometabolism has revolutionized our understanding of how immune cells, including microglia, regulate their functions. Immunometabolism posits that intracellular metabolic pathways are not merely passive suppliers of energy but are active regulators that dictate immune cell activation, polarization, and effector functions [19, 23, 24]. Microglia, like peripheral macrophages, undergo metabolic reprogramming upon activation, typically shifting from oxidative phosphorylation (OXPHOS) toward aerobic glycolysis to rapidly meet the biosynthetic and energetic demands of inflammation [25, 26]. Crucially, lipid metabolism has been identified as a particularly critical regulator of microglial biology. Alterations in lipid sensing, synthesis, and oxidation are integral to microglial inflammatory responses, phagocytic capacity, and migration [27, 28, 29, 30]. For instance, dysfunctional microglia in aging and neurodegeneration often exhibit aberrant accumulation of lipid droplets, mitochondrial deficits, and a shift in lipid utilization, which can perpetuate a pro‐inflammatory state and impair their homeostatic functions [18, 21, 31]. This intimate link between lipid metabolic imbalance and microglial dysfunction suggests that immunometabolic pathways, especially those governing lipid handling, represent a fundamental layer of regulation in neuroinflammatory diseases and a promising frontier for therapeutic intervention [20, 32, 33, 34].

A critical barrier to progress has been the lack of human‐derived mechanistic insights and the absence of a defined molecular target linking ethanol exposure to microglial metabolic dysfunction and neuroinflammation. While transcriptomic studies have hinted at broad inflammatory changes in the AUD brain, a precise, targetable pathway has remained out of reach.

Here, through a translational approach that integrates single‐cell transcriptomics from human AUD patients with validated animal and cellular models, we identify acyl‐CoA synthetase long‐chain family member 1 (ACSL1), a key enzyme in fatty acid metabolism, as a master regulator of ethanol‐induced microglial dysfunction. We demonstrate that chronic ethanol exposure triggers ACSL1 upregulation specifically in microglia, orchestrating a pathogenic cascade of lipid droplet accumulation, NLRP3 inflammasome activation, and aberrant microglia‐neuron communication via PTPRM signaling, ultimately leading to cognitive decline. Furthermore, we translate this mechanistic discovery into a potential therapeutic strategy by developing a novel dual‐targeted lipid nanoparticle system for microglia‐specific ACSL1 silencing, which effectively rescues cognitive function in chronic ethanol exposure mice.

Our study unveils ACSL1‐mediated lipoimmunometabolic reprogramming as a core pathological mechanism in AUD, providing a novel conceptual framework for understanding alcohol‐induced neurotoxicity and offering a promising targeted intervention for the cognitive deficits that accompany this disorder.

## 2 Results

### 2.1 Single‐Cell Atlas Reveals Altered Lipid Metabolism and Inflammatory Activation in Microglia of AUD Patients

**FIGURE 1:**
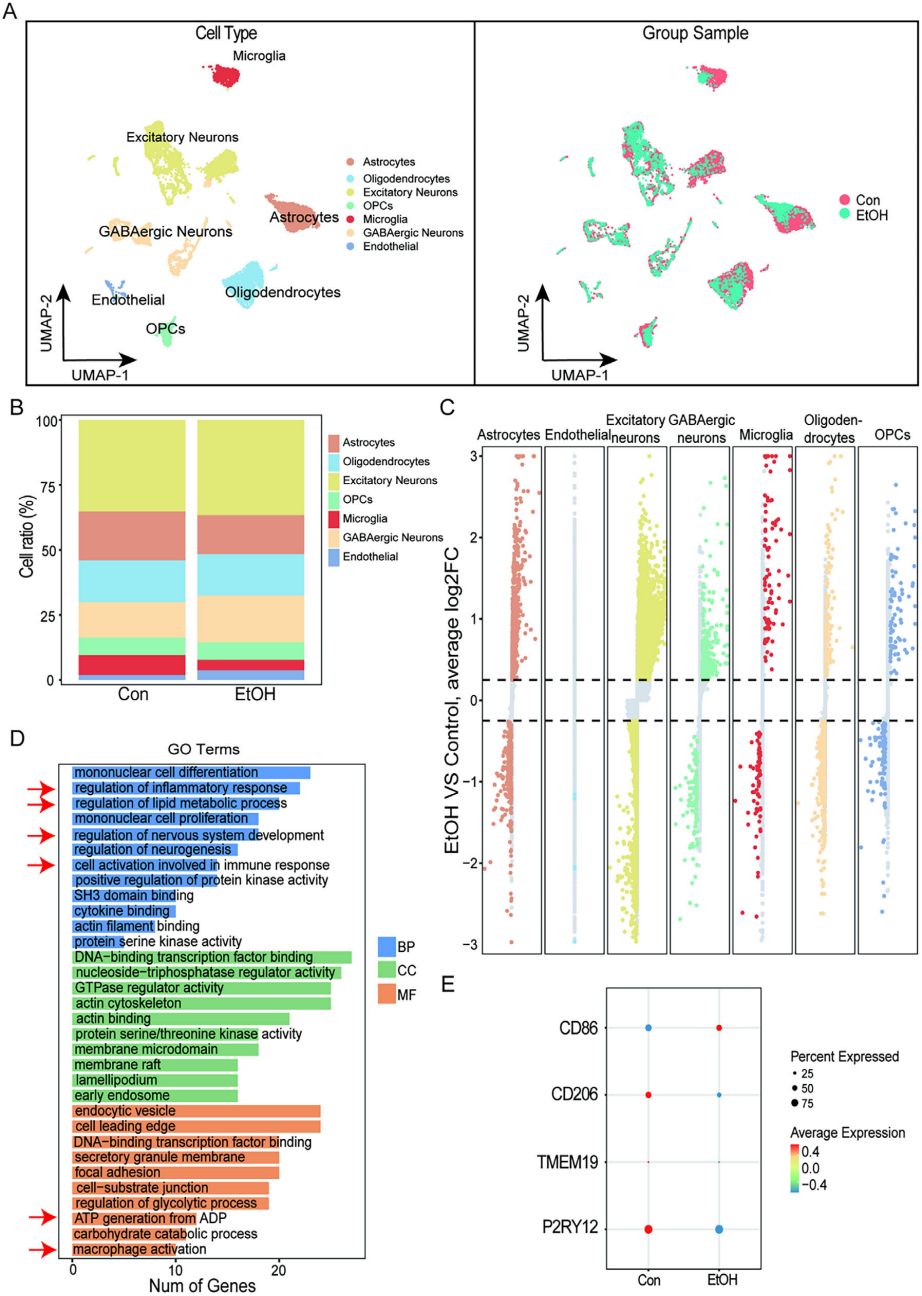
Single‐nucleus RNA sequencing reveals altered microglial states in the AUD human prefrontal cortex. (A) UMAP projection of snRNA‐seq data from the PFC of four control and three Alcohol Use Disorder (AUD) patients (GSE141552), color‐coded by the seven major identified cell types. (B) Proportional abundance of the 7 major cell types across control and AUD groups, showing a significant shift in microglial proportion. (C,D) GO biological process enrichment analysis of differentially expressed genes (DEGs) in AUD microglia, highlighting immune response, macrophage activation, and lipid metabolism pathways. (E) Validation of key microglial markers shows significant downregulation of homeostatic markers (P2RY12, TMEM119, CD206) and upregulation of the pro‐inflammatory marker CD86 in AUD microglia.

Using a single‐nucleus RNA sequencing dataset (GSE141552; 4 controls, 3 AUD patients) from the GEO database, we reanalyzed the cellular composition of the PFC in alcohol use disorder AUD. Following rigorous quality control and standardized processing, UMAP dimensionality reduction and clustering identified seven major cell types: excitatory neurons, inhibitory neurons, microglia, astrocytes, endothelial cells, oligodendrocytes, and oligodendrocyte progenitor cells (OPCs) (Figure 1A). Comparative analysis revealed no significant shifts in overall cellular proportions, consistent with previous findings [35]. Notably, the data demonstrated a downward trend in microglia abundance of AUD patients (Figure 1B). Further investigation demonstrated that differentially expressed genes in AUD microglia were significantly enriched in immune response, macrophage activation, and lipid metabolism pathways (GO analysis, Figure 1C,D). Critical marker validation confirmed significant downregulation of homeostatic microglial markers (P2RY12, TMEM119, CD206) and marked upregulation of the pro‐inflammatory marker CD86 in AUD patients (Figure 1E). These findings underscore aberrant lipid metabolism and heightened inflammatory states in microglia as key pathological features of AUD, providing new insights into neuroimmune mechanisms underlying the disorder.

### 2.2 Chronic Ethanol Exposure Induces Cognitive Impairment in a Mouse Model of Alcohol Use Disorder

**FIGURE 2:**
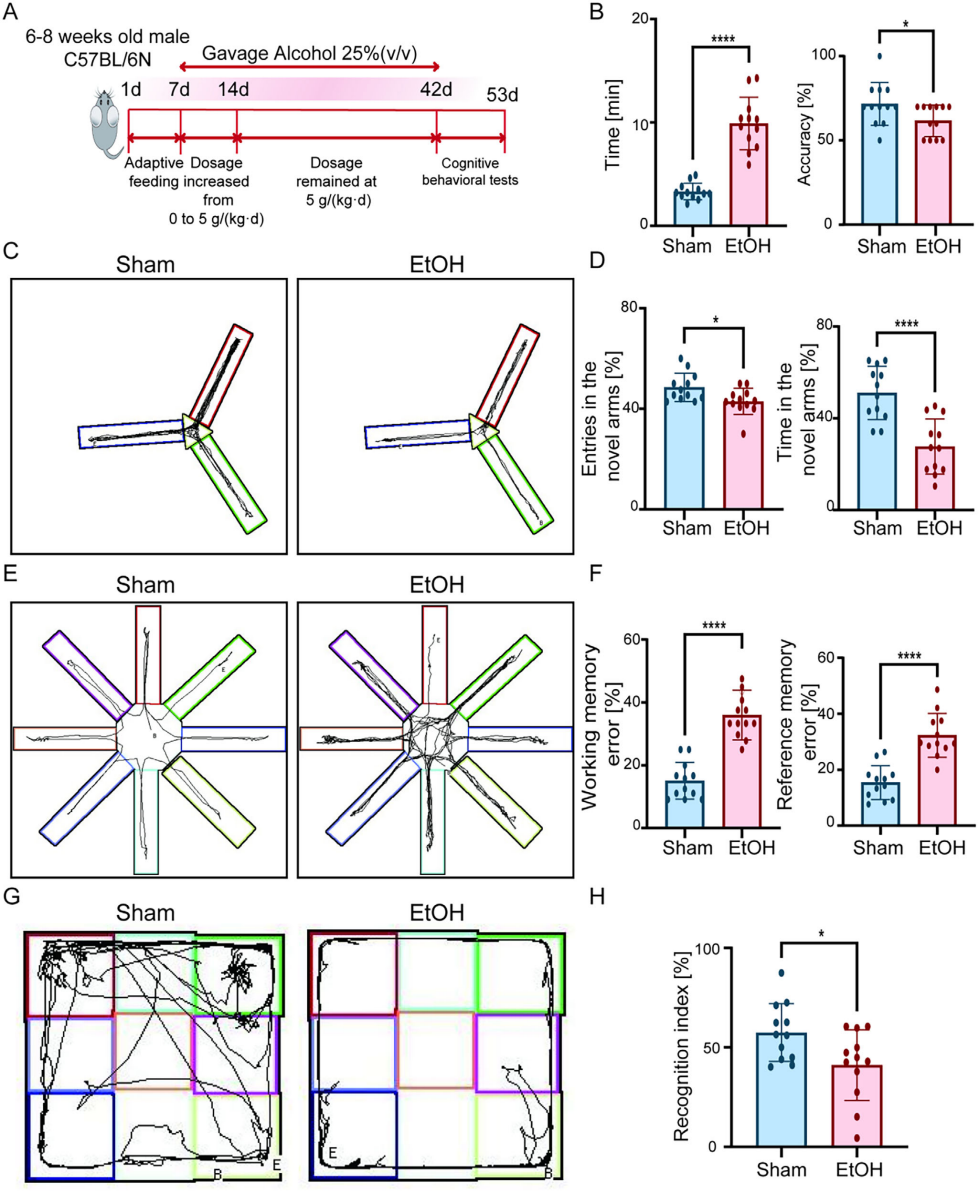
Chronic ethanol exposure induces cognitive deficits in mice. (A) Schematic of the chronic ethanol exposure paradigm in C57BL/6N mice. (B–H) Behavioral cognitive assessments show that ethanol‐exposed mice exhibit significant impairments in T‐maze spontaneous alternation (B) Y‐maze novel arm exploration (C,D), 8‐arm radial maze (E,F), and novel object recognition (G,H) compared to control mice. Data are presented as mean ± SD (*n* = 12 mice per group). ^*^ *p*< 0.05, ^****^ *p*< 0.0001; by two‐tailed unpaired t‐test.

We established a chronic ethanol exposure model using C57BL/6N mice (Figure 2A). Behavioral assessments demonstrated that ethanol‐exposed mice exhibited significantly prolonged stay time and reduced accuracy in the T‐maze test (Figure 2B). In the Y‐maze test, both the time spent and the number of entries into the novel arm were significantly decreased (Figure 2C,D). Furthermore, the eight‐arm maze test revealed significantly increased working memory error and reference memory error rates in the ethanol group (Figure 2E,F). The novel object recognition (NOR) test also showed a significant decline in the recognition index (Figure 2G,H). To rule out potential confounding effects of general motor function on cognitive performance, we assessed the average locomotor speed during the behavioral tests. Chronic ethanol exposure did not significantly alter locomotor speed in mice (Figure S1A). These results collectively indicate that chronic ethanol exposure induces significant cognitive deficits in mice.

### 2.3 Chronic Ethanol Exposure Triggers Neuroinflammatory Response in Mouse Prefrontal Cortex and Microglial Cells

**FIGURE 3:**
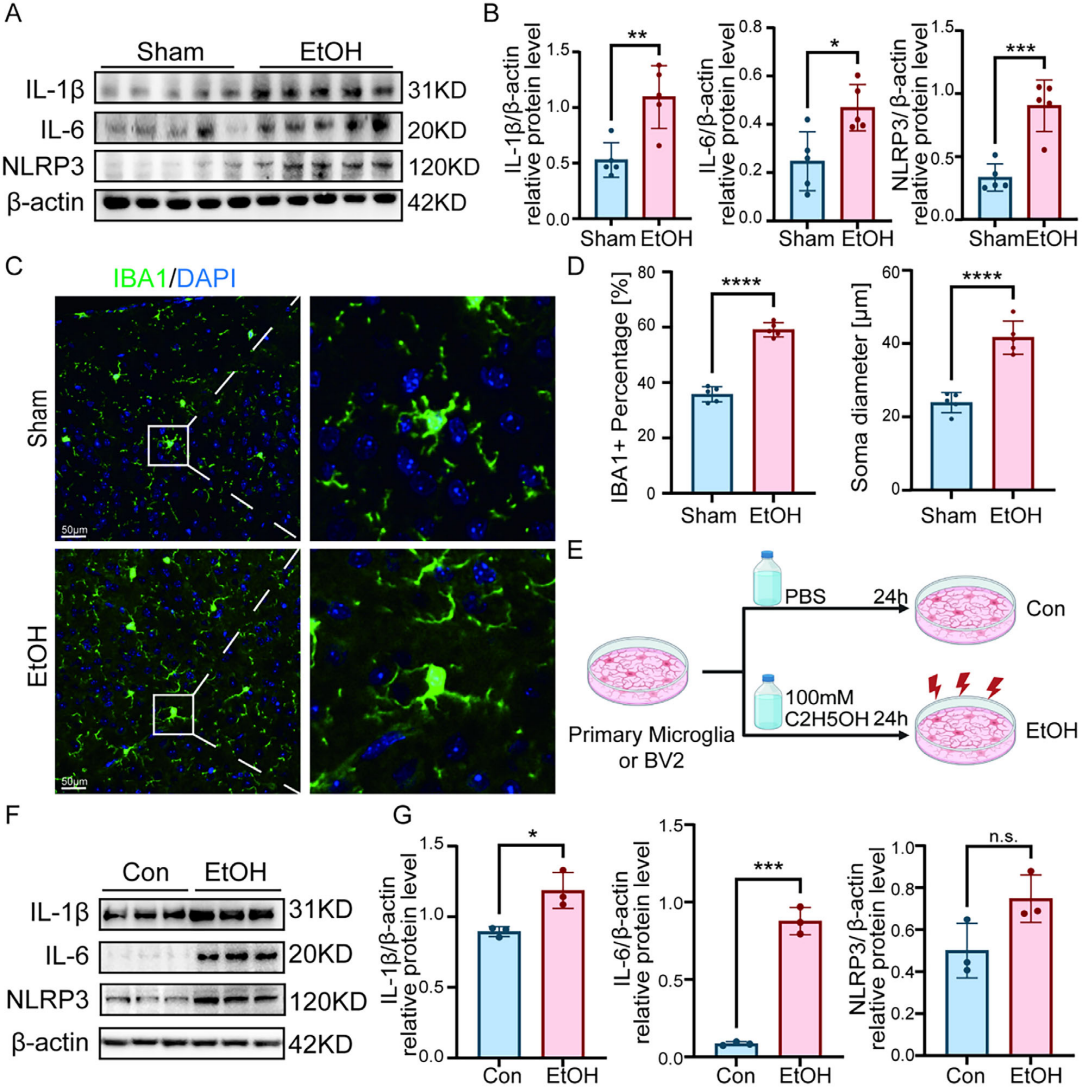
Ethanol induces neuroinflammation in vivo and in vitro. (A,B) Western blot analysis of, IL‐1β, IL‐6 and NLRP3 protein levels in the PFC of control and ethanol‐exposed mice. (C,D) Chromogenic immunohistochemistry staining and quantification of IBA1^+^ microglia in the mouse PFC, showing increased microglial density per unit area and enlarged cell body area in the ethanol group. Five randomly selected fields per mouse. (E) Schematic diagram of the chronic ethanol exposure model in primary and BV2 microglial cells. (F,G) Western blot analysis confirms upregulation of IL‐1β, IL‐6 and NLRP3, in ethanol‐treated primary microglia cells. Data are presented as mean ± SD. For in‐vivo studies (A–D): *n* = 5 mice per group. For in vitro studies (F,G): *n* = 3 independent samples per group. ^*^ *p*< 0.05, ^**^ *p*< 0.01, ^***^ *p*< 0.001, ^****^ *p*< 0.0001, n.s. nonsignificant; by two‐tailed unpaired t‐test. Scale bar, 50 µm. Figure 3F was created with BioRender.

Western blot analysis revealed a significant upregulation of key neuroinflammatory markers in the PFC of ethanol‐exposed mice, including NLRP3, IL‐1β, and IL‐6 (Figure 3A,B). Immunofluorescence analysis further demonstrated microglial activation in the PFC, characterized by increased microglial density per unit area and hypertrophic morphology in the ethanol group compared to controls (Figure 3C,D). To model these effects in vitro, we first determined a subtoxic ethanol concentration via CCK‐8 assays (Figure S1F) and subsequently established a chronic ethanol exposure model using primary and BV2 microglial cells (Figure 3E). Consistent with the in‐vivo findings, ethanol‐treated microglial cells showed significantly elevated protein levels of NLRP3, IL‐1β, and IL‐6 (Figure 3F,G; Figure S1C,D).

### 2.4 Integrated Metabolomics and Lipidomics Reveal Aberrant Lipid Metabolism in Microglia Following Chronic Ethanol Exposure

**FIGURE 4:**
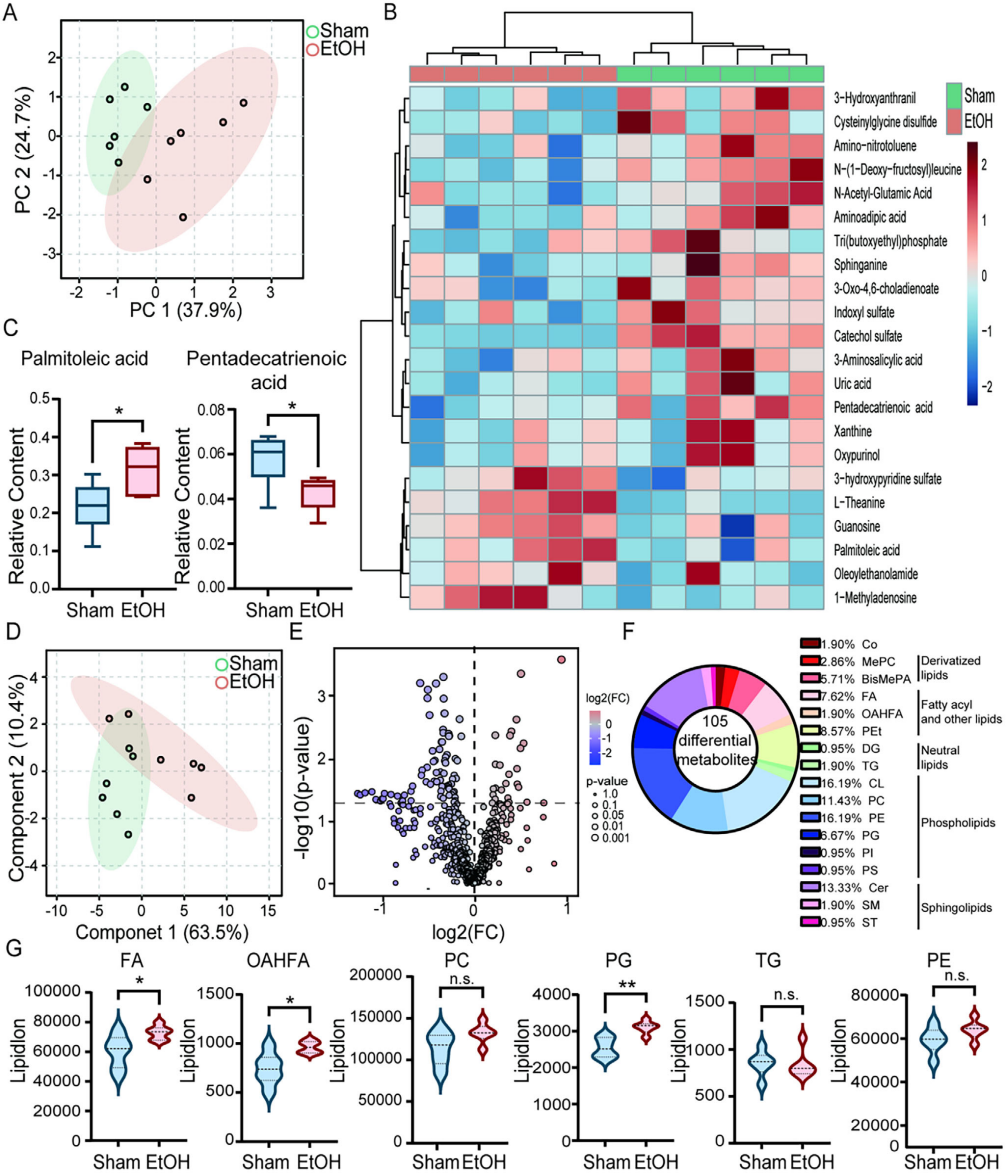
Chronic ethanol exposure disrupts lipid metabolism in the mouse PFC. (A) Principal Component Analysis (PCA) of untargeted metabolomics data from mouse PFC. (B) Heatmap of 22 significantly altered metabolites in untargeted metabolomics. (C) Relative levels of the upregulated saturated fatty acid palmitic acid and the downregulated anti‐inflammatory polyunsaturated fatty acid pentadecatrienoic acid. (D) PCA of lipidomics data from mouse PFC. (E) Volcano plot of 105 significantly dysregulated lipid species from targeted lipidomics. (F) Classification of significantly dysregulated lipid species from targeted lipidomics. (G) Relative levels of elevated lipid classes: Fatty Acyls, Fatty Acyl‐related derivatives, and Phosphatidylglycerols in the ethanol group. Data are presented as mean ± SD. *n* = 6 mice per group. ^*^ *p*< 0.05, ^**^ *p*< 0.01, n.s. nonsignificant; by two‐tailed unpaired t‐test.

Building upon our single‐cell sequencing data showing significant enrichment of lipid metabolism pathways in microglia from AUD patients (Figure 1C,D), we employed metabolomics and lipidomics approaches to investigate the impact of chronic ethanol exposure on microglial lipid metabolism. Non‐targeted metabolomic analysis of mouse (PFC) identified 22 differentially abundant metabolites, with the key lipid metabolic intermediate palmitic acid (C16:0) significantly upregulated, and the anti‐inflammatory polyunsaturated fatty acid pentadecatrienoic acid (C15:3) significantly downregulated in the ethanol‐exposed group (Figure 4A–C). Further comprehensive lipidomic profiling detected 105 differentially expressed lipid species. The ethanol group exhibited significantly elevated levels of fatty acids, acyl fatty acid derivatives, and phosphatidylglycerols (Figure 4D–G), indicating aberrant activation of lipid synthesis and storage pathways following chronic ethanol exposure.

### 2.5 Chronic Ethanol Exposure Induces Lipid Droplet Accumulation in Microglia In Vivo and In Vitro

**FIGURE 5:**
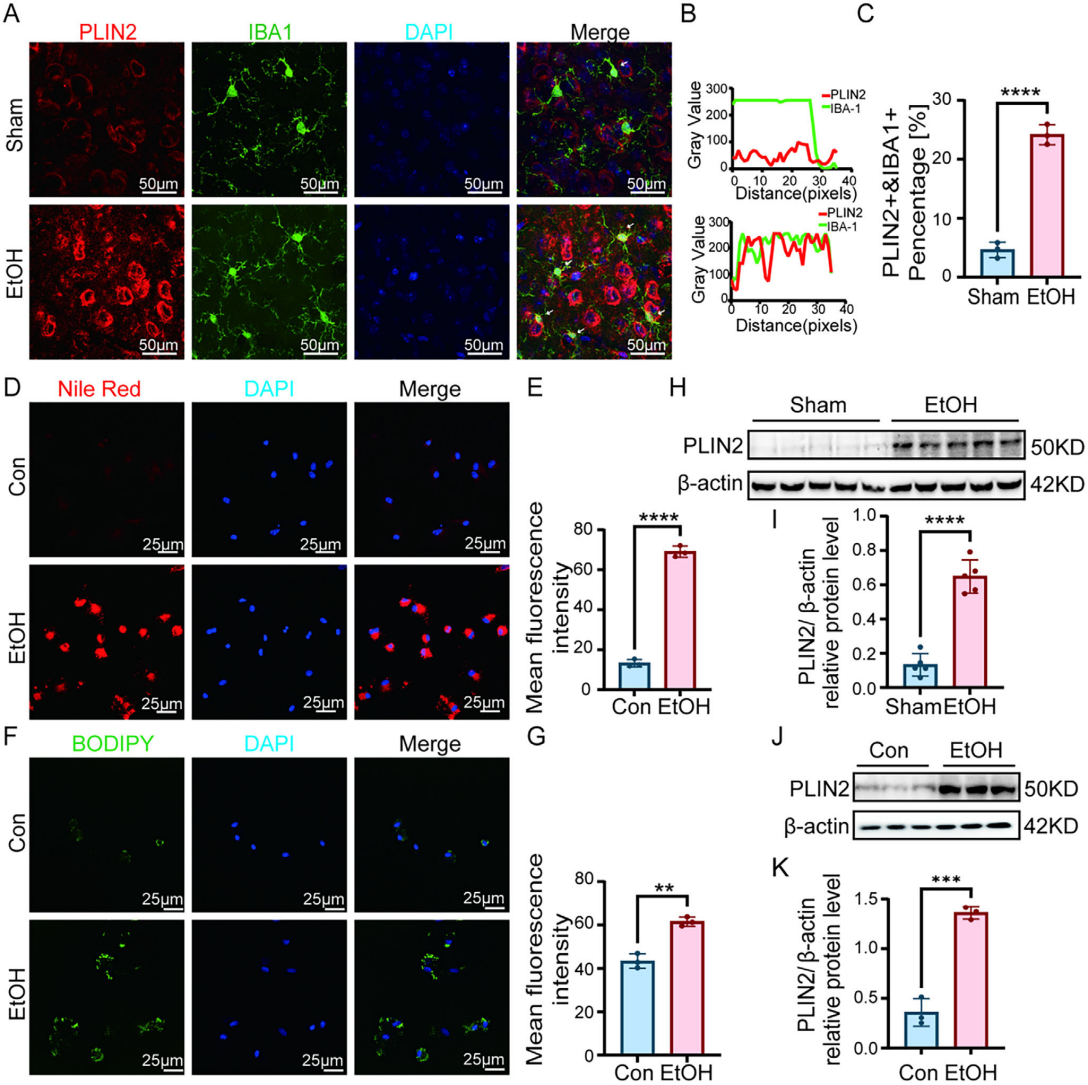
Ethanol triggers lipid droplet accumulation of microglia in vivo and vitro. (A–C) Fluorescent immunohistochemistry co‐staining for the lipid droplet protein PLIN2 and the microglial marker IBA1 in mouse PFC. Quantification shows increased PLIN2 intensity and co‐localization with IBA1^+^ cells. Five randomly selected fields per mouse. (D–G) Representative images and quantification of neutral lipid (Nile Red) and total lipid (BODIPY) staining in control and ethanol‐treated primary microglia cells. Five randomly selected fields per independent experiment. (H–K) Western blot analysis confirming significant upregulation of PLIN2 protein in both the PFC of ethanol‐exposed mice (H‐I) and in ethanol‐treated primary microglia cells (J,K). Data are presented as mean ± SD. For in‐vivo studies (A–C&H,I): *n* = 5 mice per group. For in vitro studies (D–G&J,K): *n* = 3 independent samples per group. ^**^ *p*< 0.01, ^***^ *p*< 0.001, ^****^ *p*< 0.0001; by two‐tailed unpaired t‐test. Scale bars, as shown in the figure.

Immunofluorescence analysis revealed a significant increase in the expression of the lipid droplet‐associated protein PLIN2 in microglia within the PFC of ethanol‐exposed mice. Critically, PLIN2 immunoreactivity demonstrated clear colocalization with IBA1‐positive microglial cells, indicating microglia‐specific lipid droplet accumulation in response to chronic ethanol exposure (Figure 5A–C). This finding was further corroborated in primary and BV2 microglial cell model, where both Nile Red (neutral lipids) and BODIPY (total lipids) staining showed significantly increased fluorescence intensity in ethanol‐treated cells (Figure 5D–G; Figure S1E–H). Western blot analysis consistently demonstrated upregulated PLIN2 protein expression in both animal and cellular models of chronic ethanol exposure (Figure 5H–K; Figure S1I–J).

### 2.6 ACSL1 is Uniquely Upregulated in Microglia in Response to Chronic Ethanol Exposure

**FIGURE 6:**
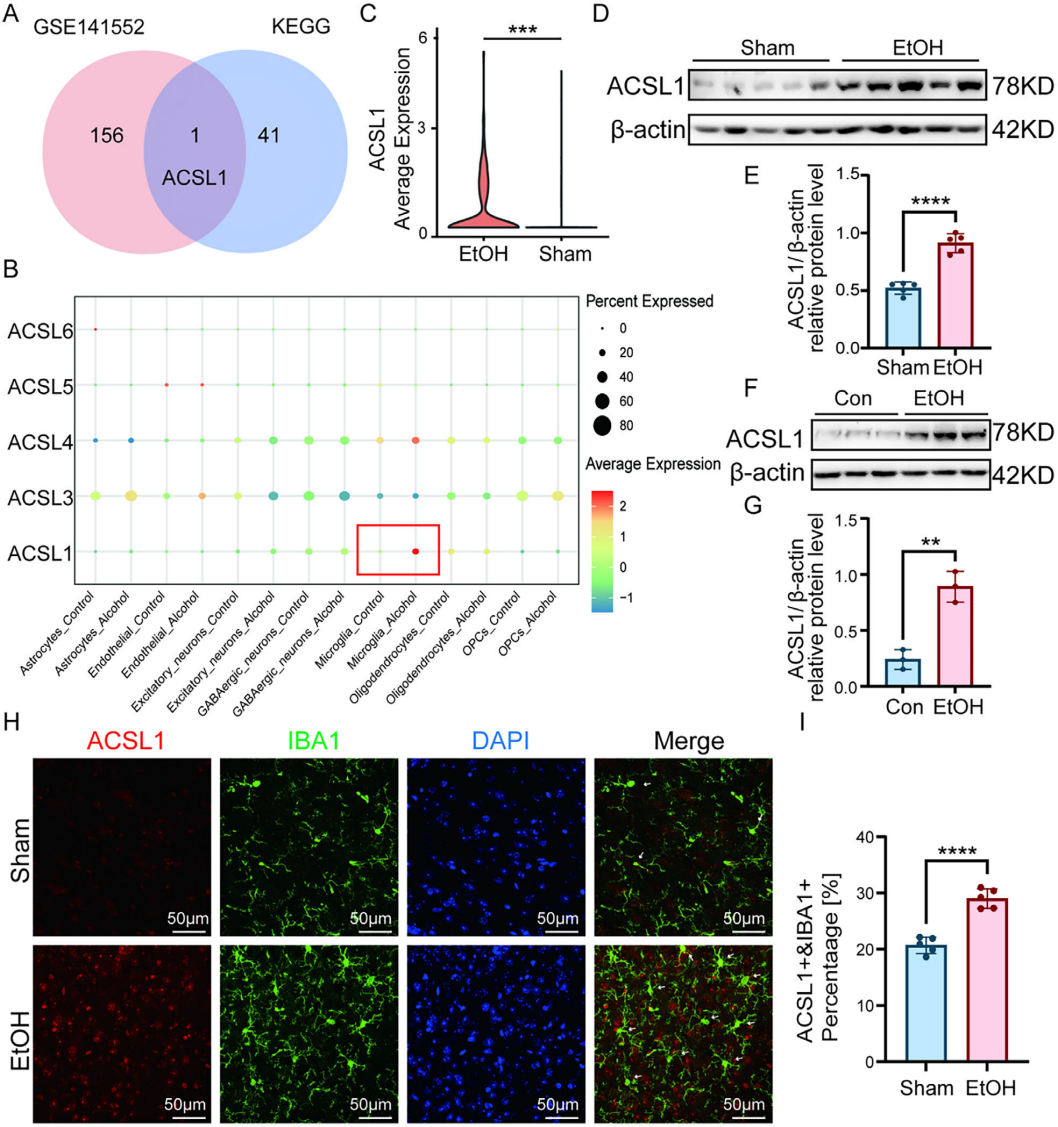
ACSL1 is identified as a key ethanol‐induced regulator in microglia. (A) Venn diagram intersection of AUD microglial DEGs and KEGG lipid metabolism gene set, identifying ACSL1. (B,C) snRNA‐seq expression of ACSL family members (ACSL1, 3‐6) across all cells (B) and specifically in microglia (C), showing specific upregulation of ACSL1 in AUD microglia. (D–G) Western blot analysis confirms ACSL1 protein is upregulated in the PFC of ethanol‐exposed mice (D,E) and in ethanol‐treated primary microglia cells (F,G). (H,I) Fluorescent immunohistochemistry staining and quantification shows specific enrichment of ACSL1 in IBA1^+^ microglia in the PFC of ethanol‐exposed mice. Five randomly selected fields per mouse. Data are presented as mean ± SD. For in vivo studies (D,E&H,I): *n* = 5 mice per group. For in‐vitro studies (F,G): *n* = 3 independent samples per group. ^**^ *p*< 0.01, ^***^ *p*< 0.001, ^****^ *p*< 0.0001; by two‐tailed t‐test. Scale bars, as shown in the figure.

Intersection analysis of differentially expressed genes in microglia from AUD patients (Figure 1C) with KEGG lipid metabolism pathway gene sets revealed selective enrichment of ACSL1, which was specifically overexpressed in the disease group (Figure 6A). Further interrogation of single‐cell sequencing data demonstrated that among ACSL family members (ACSL1, 3–6), only ACSL1 showed significant upregulation in patient microglia (Figure 6B,C), suggesting its unique functional role in alcohol‐related pathology. Western blot analysis confirmed significantly elevated ACSL1 protein expression in the PFC of chronic ethanol‐exposed mice, primary and BV2 microglial cells compared to controls (Figure 6D–G; Figure S1M–P). Immunofluorescence studies further demonstrated specific enrichment of ACSL1 within microglia in the PFC of ethanol‐exposed mice (Figure 6H,I), providing compelling evidence for microglia‐specific ACSL1 induction in response to chronic ethanol exposure.

### 2.7 ACSL1^+^ Microglia Subpopulation Drives Neuroinflammation and Lipid Accumulation in Alcohol Use Disorder

**FIGURE 7:**
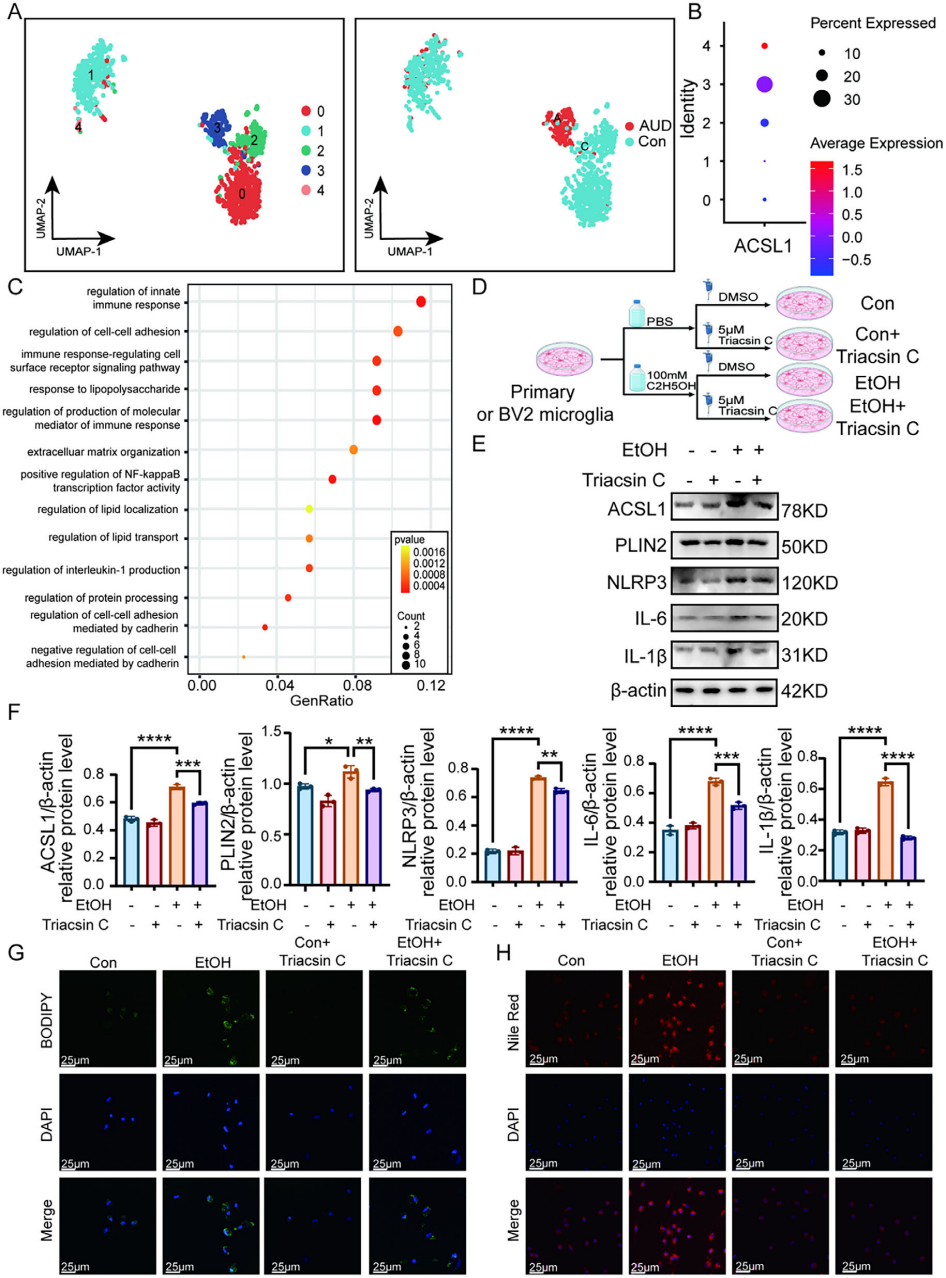
Inhibition of ACSL1 ameliorates ethanol‐induced lipid accumulation and inflammation. (A) UMAP of microglial subclustering from human snRNA‐seq data, identifying an ACSL1^+^ subpopulation (Cluster 3). (B,C) GO enrichment analysis of genes positively correlated (Pearson R>0.3) with ACSL1 expression within microglia, highlighting neuroinflammation and lipid droplet pathways. (D) Schematic of Triacsin C (ACSL1 inhibitor) treatment in the cell model. (E,F) Western blot analysis shows Triacsin C treatment reverses ethanol‐induced upregulation of ACSL1, inflammatory markers NLRP3, IL‐1β, IL‐6, and PLIN2 in primary microglia. (G,H) Quantification of Nile Red (neutral lipid) and BODIPY (total lipid) fluorescence shows Triacsin C reduces ethanol‐induced lipid accumulation in primary microglia cells. Five randomly selected fields per independent experiment. For in‐vitro studies (E–H): n = 3 independent samples per group. Data are presented as mean ± SD. ^*^ *p*< 0.05, ^**^ *p*< 0.01, ^****^ *p*< 0.0001; by one‐way ANOVA with Tukey's post hoc test. Scale bars, as shown in the figure. Figure 7D was created with BioRender.

Unsupervised clustering of microglia from the GSE141552 dataset identified four distinct subpopulations. Cluster 3, characterized by significant high expression of ACSL1, was defined as the ACSL1^+^ subpopulation (Figure 7A). GO enrichment analysis of this ACSL1^+^ subpopulation revealed that genes significantly positively correlated with ACSL1 (Pearson R > 0.3) were enriched in pathways related to neuroinflammation, cell adhesion, and lipid droplet transport (Figure 7B,C).

To functionally characterize ACSL1, we treated chronic ethanol‐exposed BV2 cells with the ACSL1 inhibitor Triacsin C (Figure 7D). Triacsin C treatment significantly suppressed protein expression levels of ACSL1, inflammatory markers (NLRP3, IL‐1β, IL‐6), and PLIN2 in ethanol‐exposed cells (Figure 7E,F; Figure S2A,B). Concurrently, both Nile Red (neutral lipids) and BODIPY (total lipids) fluorescence intensity in primary and BV2 microglial cells were markedly reduced following ACSL1 inhibition (Figure 7G,H; Figure S2C,D).

### 2.8 ACSL1^+^ Microglia Drive Neuroinflammation via Enhanced PTPRM‐Mediated Cellular Crosstalk in Alcohol Use Disorder

**FIGURE 8:**
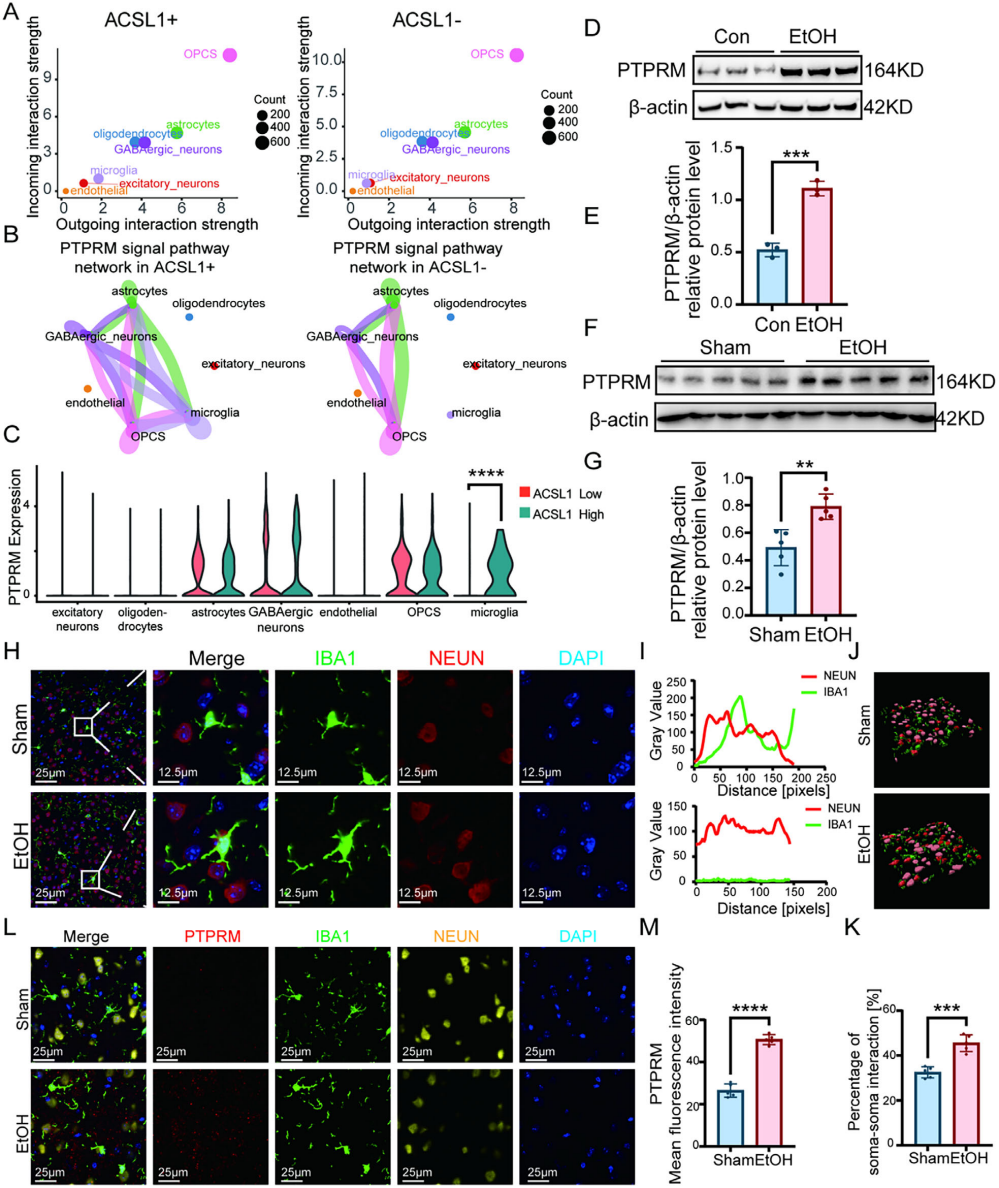
ACSL1+ microglia exhibit enhanced PTPRM‐mediated communication with neurons. (A,B) CellChat analysis predicts stronger outgoing PTPRM‐PTPRM homophilic interaction signaling from the ACSL1^+^ microglial subpopulation, primarily via autocrine communication and interactions with excitatory neurons. (C) snRNA‐seq expression of PTPRM is elevated in the human ACSL1^+^ microglial subcluster. (D–G) Western blot analysis confirms PTPRM protein is upregulated in ethanol‐treated primary microglia cells (D,E) and in the PFC of ethanol‐exposed mice (F,G). (H–K) Representative chromogenic immunohistochemistry images and quantification of IBA1^+^ microglia and NeuN^+^ neurons show increased microglia‐neuron contact frequency in the PFC of ethanol‐exposed mice. Co‐location analysis of representative images (I) and percentage of soma‐soma interaction between microglia and neurons for all neuronal populations in the imaging field (J,K). (L,M) Chromogenic immunohistochemistry intensity quantification confirms increased PTPRM expression in both IBA1^+^ microglia and NeuN^+^ neurons in the ethanol group. Five randomly selected fields per mouse. Data are presented as mean ± SD. For in vivo studies (F,G&H–M): *n* = 5 mice per group. For in‐vitro studies (D‐E): *n* = 3 independent samples per group. ^**^ *p*< 0.01, ^***^ *p*< 0.001, ^****^ *p*< 0.0001; by two‐tailed unpaired t‐test. Scale bars, as shown in the figure.

CellChat algorithm analysis comparing ligand‐receptor interaction networks between ACSL1^+^ and ACSL1^−^ microglia revealed significantly enhanced communication strength in the PTPRM‐PTPRM homodimer signaling pathway within the ACSL1^+^ subpopulation. This enhanced signaling primarily mediated autocrine microglial communication and interactions with excitatory neurons (Figure 8A,B). Single‐cell sequencing data demonstrated significantly elevated PTPRM mRNA expression in ACSL1^+^ microglia compared to controls (Figure 8C). Consistent with this finding, chronic ethanol exposure significantly upregulated PTPRM protein levels in primary, BV2 cells and mouse prefrontal cortex (Figure 8D–G; Figure S2E,F).

Immunofluorescence analysis revealed increased contact frequency between IBA1^+^ microglia and NeuN^+^ neurons in the PFC of ethanol‐exposed mice (Figure 8H–K. Furthermore, PTPRM expression was significantly upregulated in both IBA1^+^ microglia and NeuN^+^ neurons in the PFC of ethanol‐treated mice (Figure 8L,M), suggesting enhanced microglia‐neuron interactions mediated by PTPRM signaling. To investigate the role of PTPRM in chronic ethanol exposure, we generated a PTPRM knockdown model in primary microglia using siRNA (Figure S2G,H). Western blot analysis demonstrated that knockdown of PTPRM significantly attenuated the upregulation of inflammatory markers elicited by ethanol treatment (Figure S2I,J).

### 2.9 Engineering Dual‐Targeted Lipid Nanoparticles for Microglia‐Specific ACSL1 Silencing in Alcohol Use Disorder

**FIGURE 9:**
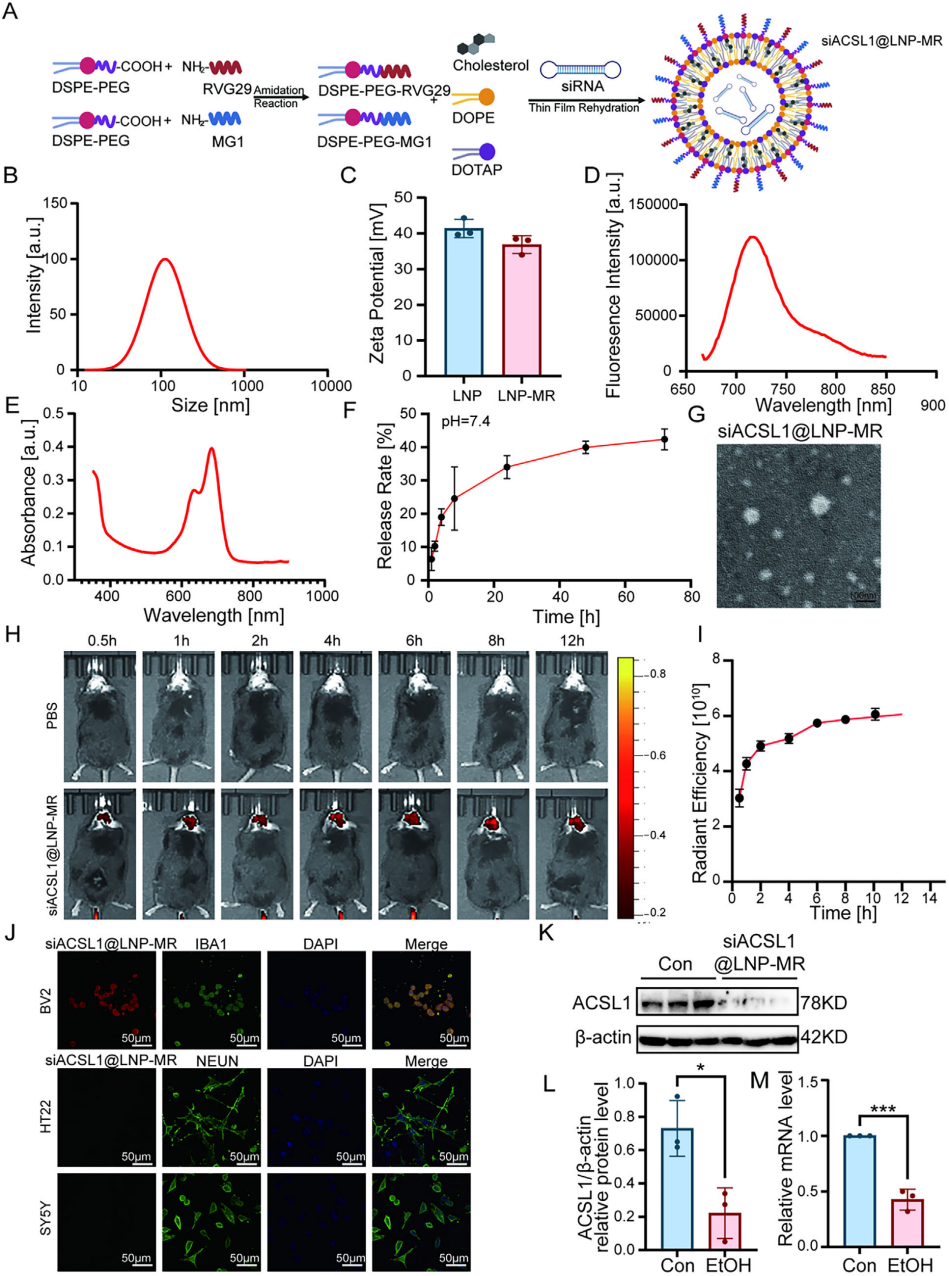
Characterization of a dual‐targeting nanoparticle for delivering ACSL1 siRNA to microglia. (A) Schematic of the siACSL1@LNP‐MR nanoparticle, engineered with RVG29 peptide for blood‐brain barrier penetration and MG1 peptide for microglial targeting. (B‐C) Characterization of nanoparticle size distribution (B) and zeta potential (C), showing successful surface modification. (D,E) Fluorescence emission spectrum (D) and UV–vis absorption spectrum (E) confirm the successful conjugation of the Cy5.5‐labeled targeting peptides to the nanoparticle surface. (F) In vitro siRNA release profile from the nanoparticle in PBS (pH 7.4) over 72 h, demonstrating a controlled release kinetics. Data are representative of at three independent experiments (B, C, F) or scans (D, E). (G) Transmission electron microscopy (TEM) image reveals uniform spherical morphology of siACSL1@LNP‐MR with an average diameter of approximately 100 nm. (H,I) In vivo fluorescence imaging of mouse heads at indicated time points after tail vein injection of Cy5.5‐labeled nanoparticles shows time‐dependent accumulation and peak brain fluorescence intensity between 8–12 h post‐injection. Data are representative of at three independent experiments. (J) Representative images of Cy5.5‐labeled siACSL1@LNP‐MR targeted BV2 cells. (K–M) Western blot (K,L) and qRT‐PCR (M) analysis confirm ACSL1 protein is downregulated in siACSL1@LNP‐MR‐treated BV2 cells. For in‐vivo and vitro studies (H,J–L): *n* = 3 independent samples per group. ^*^ *p*< 0.05, ^***^ *p*< 0.001; by two‐tailed unpaired t‐test. Scale bars, as shown in the figure.

Given the pivotal role of ACSL1 in AUD, we developed a novel dual‐targeted lipid nanoparticle system (siACSL1@LNP‐MR) for precision delivery of ACSL1 siRNA to microglia in the context of alcohol use disorder. The nanoparticle surface was functionalized with RVG29, a blood‐brain‐barrier (BBB) targeting peptide, and MG1, a microglia‐specific targeting peptide, enabling efficient BBB penetration and selective microglial engagement (Figure 9A). Characterization studies revealed nanoparticles with a uniform size of 114.84 ± 1.26 nm and a zeta potential of 36.87 ± 2.47 mV, notably lower than non‐targeted blank LNPs (41.06 ± 2.84 mV) (Figure 9B,C). Fluorescence and ultraviolet spectroscopy confirmed successful surface modification, showing characteristic peaks at 716 nm and 690 nm respectively, corresponding to the conjugated Cy5.5 label (Figure 9D,E). siRNA release kinetics demonstrated rapid payload release under physiological conditions (pH 7.4), with approximately 45% of siRNA released within 72 h (Figure 9F), indicating favorable release characteristics for therapeutic application. Transmission electron microscopy (TEM) imaging revealed that the siACSL1@LNP‐MR nanoparticles exhibited a relatively uniform spherical morphology with an average diameter of approximately 100 nm, which is consistent with the results obtained from dynamic light scattering‐based particle size analysis (Figure 9G).

To evaluate the ability of the nanoparticles to cross the blood‐brain barrier (BBB) and accumulate in the brain in vivo, Cy5.5‐labeled siACSL1@LNP‐MR was administered to mice via tail vein injection. Fluorescence intensity in the head region was monitored at 0.5, 1, 2, 4, 6, 8, and 12 h post‐injection. The results demonstrated a time‐dependent increase in fluorescence signal, which peaked between 8 and 12 h after administration, indicating effective BBB penetration and sustained accumulation of the nanoparticles in the brain (Figure 9H,I). To validate the cellular targeting and functional efficacy of the nanoparticles, we first examined their uptake across different neural cell types using fluorescent labeling. Cy5.5‐labeled nanoparticles were specifically internalized by BV2 microglial cells, showing significant co‐localization with the microglial marker IBA1. In contrast, uptake was negligible in neuronal HT22 and SY5Y cells (Figure 9J). Western blot analysis confirmed effective downregulation of ACSL1 protein expression by the siRNA‐loaded nanoparticles in BV2 cells (Figure 9K,I). Collectively, these findings demonstrate the microglia‐specific targeting and functional ACSL1 modulation mediated by our nanoparticle system.

### 2.10 Silencing Microglial ACSL1 Reverses Ethanol‐Induced Cognitive Decline by Mitigating Neuroinflammation and Lipid Accumulation

**FIGURE 10:**
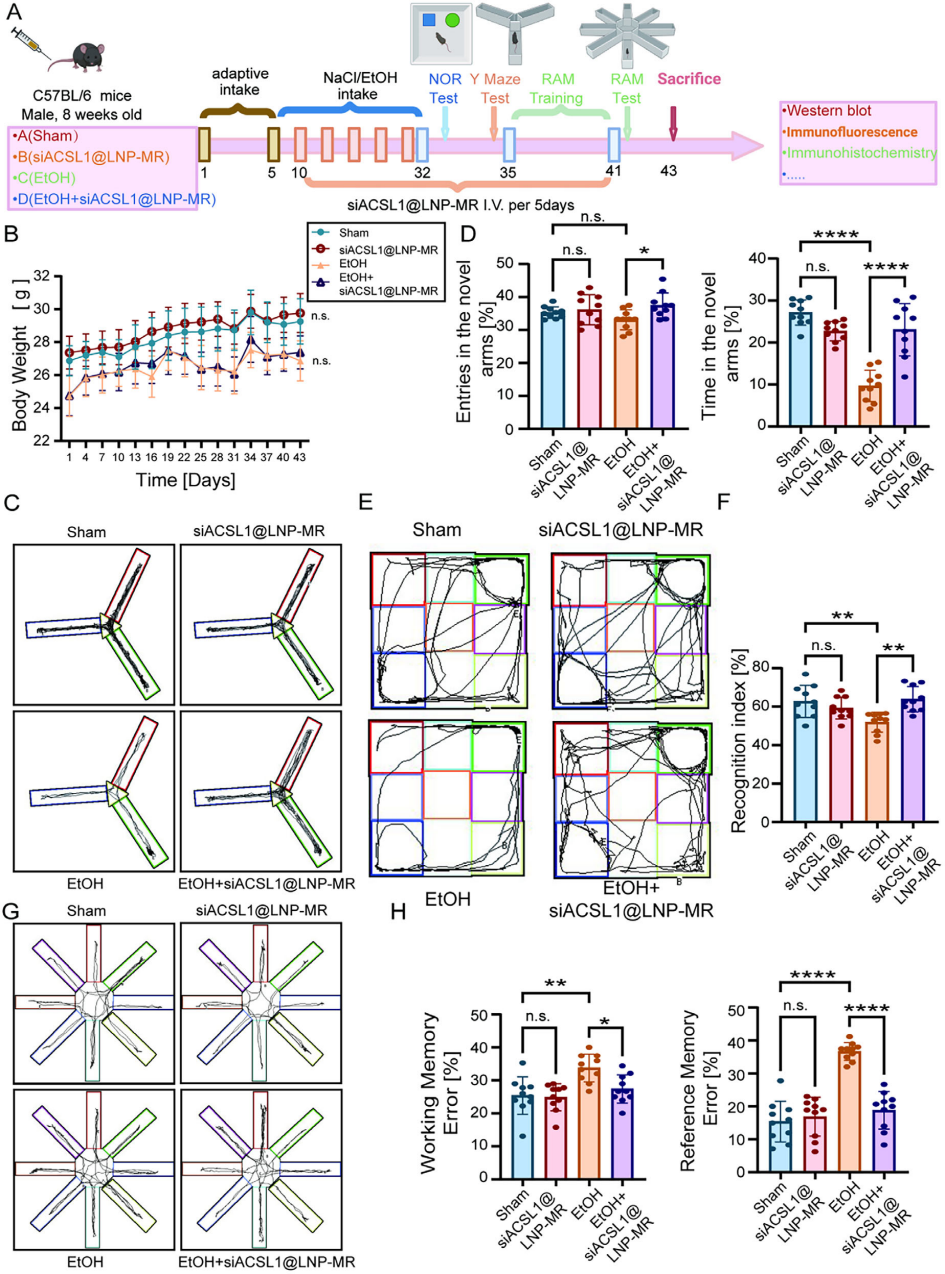
Targeted Silencing of Microglial ACSL1 Reverses Cognitive Deficits in a Mouse Model of Chronic Ethanol Exposure. (A) Schematic diagram of the experimental timeline for chronic ethanol exposure and siACSL1@LNP‐MR administration. (B) Body weight of mice throughout the experimental period across the indicated groups (*n* = 10 mice per group). (C, D) Y‐maze test performance. (C) Percentage of time spent in the novel arm. (D) Percentage of entries into the novel arm (*n* = 10 mice per group). (E, F) Novel object recognition test. (E) Representative tracking plots of mice during the test. (F) Recognition index quantified for each group (*n* = 10 mice per group). (G, H) Eight‐arm radial maze performance. (G) Percentage of working memory errors. (H) Percentage of reference memory errors (*n* = 10 mice per group). All bar graph data are presented as mean ± SD. ^*^ *p*< 0.05, ^**^ *p* < 0.01, ^****^ *p* < 0.0001, n.s. nonsignificant; by one‐way ANOVA with Tukey's post hoc test (unless otherwise specified).

To evaluate the therapeutic potential of targeting microglial ACSL1 in alcohol‐related cognitive impairment, we established a chronic ethanol exposure mouse model and administered siACSL1@LNP‐MR via tail vein injection (Figure 10A). We first confirmed the biosafety of our therapeutic approach. siACSL1@LNP‐MR did not significantly affect mouse body weight (Figure 10B) and induced no apparent pathological alterations in major organs, including the heart, liver, spleen, lungs, and kidneys (Figure S3A), indicating a favorable biological safety profile.

We next assessed cognitive behavioral outcomes. Remarkably, siACSL1@LNP‐MR treatment effectively reversed ethanol‐induced cognitive deficits. In the Y‐maze test, ethanol‐exposed mice treated with siACSL1@LNP‐MR exhibited a significant increase in both the time spent and the number of entries into the novel arm (Figure 10C,D). In the novel object recognition test, the discrimination index, which was reduced by ethanol exposure, was significantly restored to levels comparable to the Sham group in the EtOH+siACSL1@LNP‐MR cohort (Figure 10E,F). Furthermore, in the eight‐arm radial maze, siACSL1@LNP‐MR treatment significantly reduced both working memory errors and reference memory errors elevated by chronic ethanol intake (Figure 10G,H).

**FIGURE 11:**
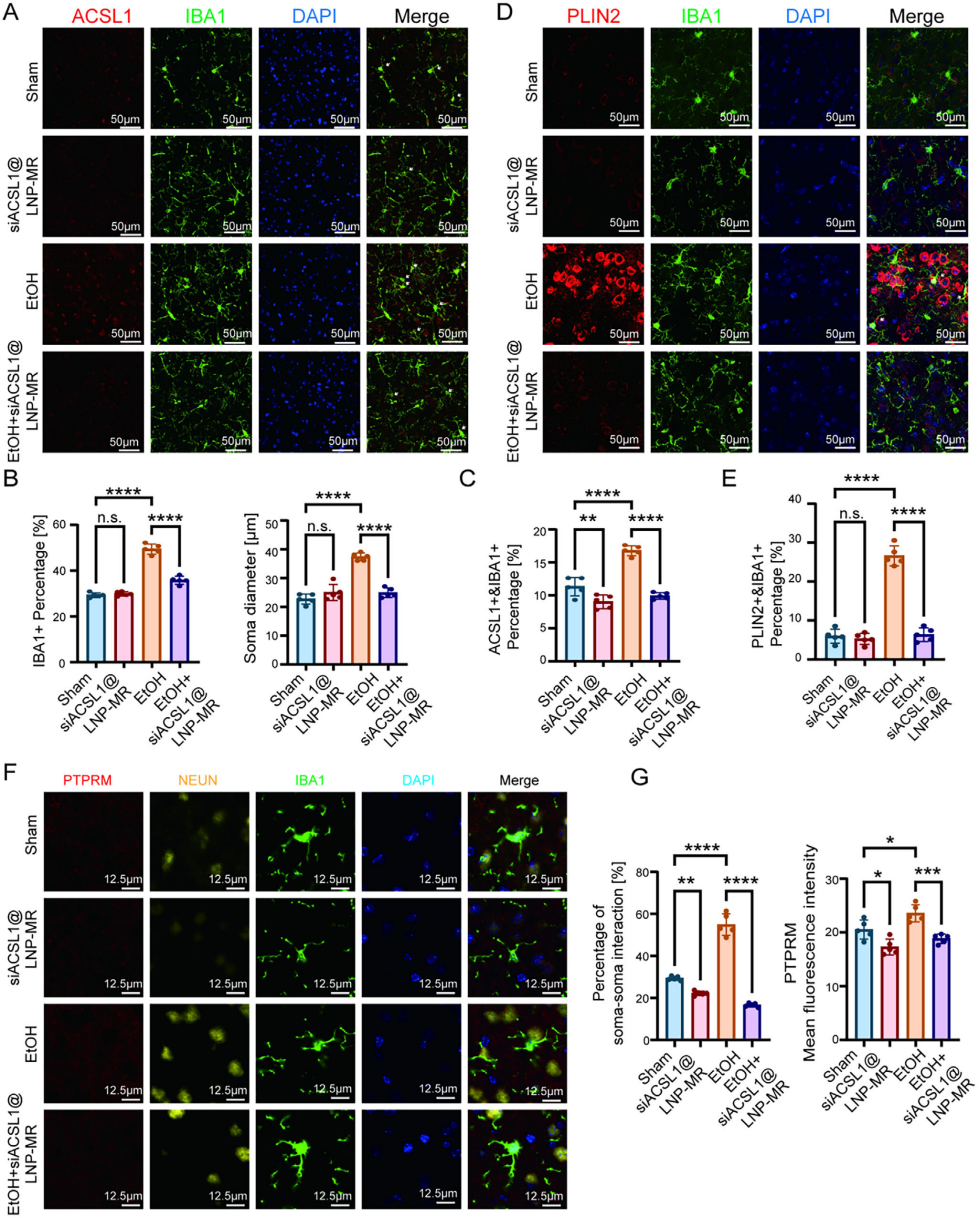
Targeted Silencing of Microglial ACSL1 Reverses Neuropathology in a Mouse Model of Chronic Ethanol Exposure. (A,B) Analysis of microglial activation in the prefrontal cortex (PFC). (A) Representative fluorescent immunohistochemistry images of ACSL1 expression in IBA1^+^ microglia. Scale bar, as shown in the figure. (B) Quantification of the percentage of IBA1^+^ unit area and microglial soma diameter (*n* = 5 mice per group). (C) Representative fluorescent immunohistochemistry images and quantification of ACSL1 expression (red) in IBA1^+^ microglia (green) in the PFC (*n* = 5 mice per group). Scale bar, as shown in the figure. (D,E) Assessment of lipid accumulation in microglia. (D) Representative fluorescent immunohistochemistry images of PLIN2 (red) in IBA1^+^ microglia (green) in the PFC (*n* = 5 mice per group). Scale bar, as shown in the figure. (E) Quantification of PLIN2 fluorescence intensity per microglia (*n* = 5 mice per group). (F,G) Analysis of microglia‐neuron interactions. (F) Representative chromogenic immunohistochemistry images of PTPRM protein levels in the PFC (*n* = 5 mice per group). (G) Representative images and quantification of the contact rate between IBA1^+^ microglia (green) and NeuN^+^ neurons (yellow)(left) and Quantification of PTPRM fluorescence intensity(right) (*n* = 5 mice per group). Scale bar, as shown in the figure. All bar graph data are presented as mean ± SD. ^*^ *p*< 0.05, ^**^ *p*< 0.01, ^***^ *p*< 0.001, ^****^ *p*< 0.0001, n.s. nonsignificant; by one‐way ANOVA with Tukey's post hoc test.

To elucidate the underlying mechanisms, we examined the PFC. Immunofluorescence analysis revealed that the ethanol‐induced microgliosis, characterized by increased percentage of IBA1^+^ cells and enlarged soma diameter, was markedly attenuated by siACSL1@LNP‐MR (Figure 11A,B). As expected, ACSL1 expression within microglia was efficiently knocked down (Figure 11C). Concomitantly, the pronounced lipid accumulation observed in microglia of ethanol‐fed mice was significantly reduced following siACSL1@LNP‐MR treatment (Figure 11D,E). We also investigated the involvement of PTPRM, a protein implicated in microglia‐neuron interactions. Notably, siACSL1@LNP‐MR treatment significantly suppressed the ethanol‐induced upregulation of PTPRM and consequently inhibited the increased rate of microglia‐neuron contacts (Figure 11F,G).

## 3 Discussion

AUD imposes severe and often lasting cognitive deficits, yet the neuroimmune mechanisms contributing to these impairments remain poorly elucidated. Here, we identify ACSL1‐mediated lipoimmunometabolic reprogramming of microglia as a central mechanism driving ethanol‐induced cognitive dysfunction, revealing a previously unappreciated pathogenic pathway in AUD.

Our multiomics approach, initiated with re‐analysis of human single‐nucleus RNA sequencing data, uncovered a pronounced enrichment of lipid metabolic pathways in microglia from AUD patients, coupled with a robust inflammatory activation signature. This metabolic imbalance alongside neuroinflammation dual phenotype led us to hypothesize that dysregulated lipid handling in microglia represents a critical nexus in alcohol‐related neuropathology. Using integrated metabolomic and lipidomic profiling in a preclinical model, we confirmed that chronic ethanol exposure induces substantial disruption of cerebral lipid homeostasis, marked by accumulation of fatty acids, acyl derivatives, and phosphatidylglycerols. Importantly, these alterations were accompanied by significant lipid droplet deposition specifically within microglia, both in vivo and in vitro, indicating a maladaptive metabolic shift in microglia under ethanol challenge.

The interplay between chronic alcohol exposure and dysregulated lipid metabolism is a well‐documented pathogenic feature in peripheral organs, particularly the liver, where it propels alcohol‐associated liver disease (ALD). At the heart of this metabolic dysregulation is ACSL1, an enzyme essential for activating free fatty acids into acyl‐CoAs, directing them toward β‐oxidation, phospholipid synthesis, or lipid storage [36]. Notably, rodent models of ALD consistently demonstrate that chronic alcohol consumption downregulates hepatic ACSL1 expression [36, 37, 38]. This suppression is mechanistically consequential, as hepatocyte‐specific deletion of *Acsl1* exacerbates alcohol‐induced liver injury, promoting accumulation of cytotoxic free fatty acids, lysosomal membrane permeabilization, and ultimately hepatocyte death [36]. Conversely, interventions that mitigate ALD, such as treatment with umbelliferone, scopoletin, or extracts from *Hovenia dulcis* or *Rhododendron nivale*, often confer protection by upregulating ACSL1 and restoring fatty acid oxidative capacity [38, 39, 40].

While these findings clearly establish a protective role for ACSL1 in the periphery where its loss exacerbates lipotoxicity and cell death, ACSL1 upregulation in the PFC of AUD patients, and in ethanol‐exposed mice and microglia (Figure 6) observed in the current study, reveals a striking and paradoxical cell‐type‐specific dichotomy.

This contrast underscores a fundamental biological principle, the functional impact of a metabolic enzyme is highly dependent on cellular context and metabolic state. In specialized lipid‐processing hepatocytes, ACSL1 directs fatty acids toward catabolic β‐oxidation, supporting energy production and mitigating lipotoxicity. In microglia, however, its role appears divergent. Within the immunometabolic milieu of activated microglia, ACSL1 upregulation may divert fatty acids toward anabolic pathways, promoting synthesis of inflammatory lipids or contributing to lipid droplet formation, a hallmark of dysfunctional, pro‐inflammatory microglia seen in neurodegeneration [41]. Our observation that the ACSL1^+^ microglial subcluster is enriched for pathways such as lipid droplet assembly (Figure 7B,C) strongly supports this notion. Thus, in microglia, ACSL1 may act not protectively, but as a pathogenic driver of alcohol‐induced immunometabolic reprogramming, lipid droplet accumulation, and neuroinflammation.

Functional studies using pharmacological inhibition confirmed that ACSL1 is causative in lipid droplet accumulation, NLRP3 inflammasome activation, and pro‐inflammatory cytokine release. Moreover, single‐cell communication analysis revealed that ACSL1^+^ microglia display enhanced autocrine and paracrine signaling, particularly via PTPRM‐PTPRM homophilic interactions, likely amplifying neuroinflammatory responses and disrupting microglia‐neuron communication. This provides a mechanistic link between microglial metabolic reprogramming and neural circuit dysfunction, offering a plausible basis for the working and reference memory deficits observed in our behavioral assays.

Our findings situate AUD within an expanding framework of neurological disorders driven by microglial metabolic dysfunction. Genetic and multi‐omics studies have firmly implicated ACSL1 in Alzheimer's disease (AD). Mendelian randomization analysis identified *Acsl1* as causally linked to AD (along with *Fam117a*), providing strong genetic evidence that ACSL1 variation influences disease risk [42]. Cortical transcriptomic studies from early AD patients consistently show ACSL1 upregulation alongside other key lipid metabolic genes, implicating mitochondrial fatty acid β‐oxidation and cholesterol metabolism in disease progression [43]. This upregulation is cell‐type‐specific, as single‐nucleus RNA sequencing identifies microglia as a primary site of ACSL1 dysregulation in AD [44, 45]. In this context, ACSL1 likely shunts fatty acids away from oxidation toward esterification and lipid droplet formation, a process potentially worsened by APOE4‐impaired lipid droplet autophagy [46]. A similar ACSL1‐high microglial subtype has been described in Parkinson's disease (PD), operating through a self‐amplifying positive feedback loop that drives pathogenicity [45]. This microglial pathway also exhibits sexual dimorphism: microglia from females, who are at higher AD risk, display a baseline transcriptomic profile resembling a disease‐associated state [47].

The role of ACSL1 in AD demonstrates a compelling cell‐type and context‐dependent duality, echoing the paradox we describe in AUD. In hepatocytes and adipocytes, ACSL1 is generally associated with anabolic lipid synthesis [48, 49]. However, in microglia in the context of AD or AUD, its anabolic function with promoting lipid droplet accumulation is fundamentally detrimental. This stands in stark contrast to its role in peripheral tissues and emphasizes that the functional output of a metabolic enzyme is intimately tied to the cellular environment and pathological drivers.

Our study extends this concept beyond AD by demonstrating that ACSL1 upregulation in alcohol‐activated microglia is associated with neuroinflammation and cognitive decline. These results suggest that ACSL1‐mediated immunometabolic reprogramming is a conserved mechanism underlying microglial dysfunction across diverse neurological conditions from neurodegenerative dementias to substance use disorders. The convergence of APOE4 in AD and chronic ethanol exposure in AUD on a shared ACSL1‐high microglial phenotype points to a unified pathological pathway centered on lipid metabolic dysregulation in brain immune cells.

While our work establishes a role for ACSL1 in alcohol‐induced microglial immunometabolism, several important considerations and future directions emerge from our findings. A key observation is the differential microglial population dynamics between species. Microglial numbers increased in our mouse model, whereas human AUD postmortem samples showed no significant change in overall proportion, consistent with the original dataset [19]. Beyond limitations in sample size and the impact of interindividual and cross‐species variability, we interpret this discrepancy as likely reflecting distinct disease stages: an active, proliferative phase captured in the rodent model vs. represented in human tissue [50]. Critically, despite this divergence in cellular abundance, ACSL1‐mediated immunometabolic phenotype was conserved, underscoring its fundamental role across models. Future studies employing expanded human cohorts and longitudinal animal models will be essential to resolve the temporal dynamics of microglial responses across AUD progression.

Our rodent findings in the mPFC, particularly the prelimbic (PL) and infralimbic (IL) subregions, align with human neuroimaging and postmortem studies implicating medial PFC circuits in AUD‐related cognitive impairment [2, 12, 14]. Future studies should dissect subregion‐specific contributions, such as the role of dorsolateral PFC in working memory vs. orbital PFC in reward processing, to refine our understanding of circuit‐level pathology in AUD. While we focused on PFC microglia, it remains unclear whether similar mechanisms operate in other AUD‐affected regions such as the hippocampus or amygdala. Given the established role of hippocampal dysfunction in AUD‐related memory deficits [51, 52, 53], future work should assess whether ACSL1 upregulation and lipid droplet accumulation occur consistently across neural circuits impacted by alcohol. Additionally, the temporal dynamics of ACSL1 induction and lipid droplet formation require further investigation to determine whether these represent early adaptive or late degenerative responses. A systematic time‐course analysis tracking ACSL1 expression, lipid droplet accumulation, and neuroinflammatory activation could clarify the sequence of events and strengthen causal inference. Such studies may identify critical windows for intervention and elucidate whether ACSL1 upregulation initiates or amplifies neuroinflammation. Lastly, although our nanoparticle approach shows encouraging targeting efficiency, its transition to human applications will require optimization in scalability, stability, and immunogenicity. Comparative studies of different ACSL1 inhibition strategies including small‐molecule inhibitors and genetic tools could help identify the most clinically viable approach.

In conclusion, our work establishes ACSL1‐dependent microglial lipoimmunometabolic reprogramming as a central pathological mechanism in alcohol‐induced cognitive decline. By integrating human transcriptomics, rodent models, multi‐omics, and nanotherapeutic engineering, we provide a comprehensive framework for understanding how alcohol reprograms microglial metabolism to promote neuroinflammation and neural dysfunction. These findings nominate ACSL1 as a promising therapeutic target for AUD and potentially other neuroinflammatory disorders characterized by lipid‐laden microglia.

## 4 Materials and Methods

### 4.1 Ethical Compliance

All human data were obtained from the publicly available Gene Expression Omnibus (GEO) dataset GSE141552. Animal experiments were conducted in accordance with the National Institutes of Health Guide for the Care and Use of Laboratory Animals and approved by the Institutional Animal Care and Use Committee (IACUC) of China Medical University.

### 4.2 Human Single‐Nucleus RNA Sequencing Data Analysis

Preprocessed single‐nucleus RNA sequencing data from PFC samples of 4 control and 3 AUD patients (GSE141552) were downloaded from the GEO database. Based on established anatomical consensus and prior literature describing this dataset, the sample is positioned to encompass the medial prefrontal cortex (mPFC), including key subregions such as the anterior cingulate cortex (ACC) and orbitofrontal cortex (OFC)‐areas integral to emotion regulation, decision‐making, and cognitive control. Quality control, normalization, clustering, and differential expression analysis were performed using Seurat (v4.3.0). Cells with fewer than 200 genes or >10% mitochondrial reads were excluded. Uniform Manifold Approximation and Projection (UMAP) was used for dimensionality reduction. Cell types were annotated using canonical markers. Gene Ontology (GO) and KEGG pathway enrichment analyses were conducted using clusterProfiler (v4.6.2).

### 4.3 Synthesis and Characterization of siACSL1@LNP‐MR Nanoparticles

#### 4.3.1 Materials

1,2‐Dioleoyl‐3‐trimethylammonium‐propane(DOTAP), 1,2‐Dioleoyl‐sn‐glycero‐3‐phosphoethanolamine(DOPE), Cholesterol,1,2‐Distearoyl‐sn‐glycero‐3‐phosphoethanolamine‐N‐[maleimide(polyethyleneglycol)](DSPE‐PEG‐MAL), 1,2‐Distearoyl‐sn‐glycero‐3‐phosphoethanolamine‐N‐[succinimidyl carboxy(polyethylene glycol)] (DSPE‐PEG‐NHS), MG1 peptide (CHHSSSARC), RVG29 peptide (YTIWMPENPRPGTPCDIFTNSRGKRASNGC) and Cyanine5.5 (Cy5.5) were procured from Xi'an Ruixi Biotechnology Co., Ltd. The *ACSL1*‐targeting RNAi plasmid (siRNA sequence: 5′‐GGAUGCUUCUCUUACUCAAUG‐3′) was synthesized by Shenyang Mingsheng Biotechnology Co., Ltd.

#### 4.3.2 Preparation of siACSL1@LNP‐MR

DSPE‐PEG‐RVG29 was synthesized by dissolving 100 mg of DSPE‐PEG‐MAL in 3 mL of DMF, adding 1.1 equivalents of RVG29 peptide, and reacting at room temperature for 12 h. The product was then dialyzed against pure water using a 3500 Da molecular weight cut‐off dialysis bag for 24 h, followed by freeze‐drying to obtain the final conjugate.

DSPE‐PEG‐MG1 was prepared by reacting DSPE‐PEG‐NHS with the MG1 peptide in the presence of triethylamine. Briefly, 100 mg of DSPE‐PEG‐NHS was dissolved in 3 mL of anhydrous DMF. MG1 peptide (1.1 equiv.) and triethylamine (3.0 equiv.) were added and stirred until complete dissolution. The reaction proceeded at room temperature for 12 h. The mixture was then transferred into a dialysis membrane (MWCO: 1000 Da) and dialyzed against deionized water for 24 h. The retentate was collected and lyophilized to obtain DSPE‐PEG‐MG1 as a white solid.

The siRNA‐loaded liposomes (siACSL1@LNP‐MR) were prepared by dissolving DOTAP (30 mg), DOPE (15 mg), cholesterol (3.5 mg), DSPE‐PEG‐MG1 (2.5 mg), DSPE‐PEG‐RVG29 (2.5 mg), and DSPE‐PEG‐CY5.5 (1.5 mg) in 2 mL of anhydrous ethanol, followed by addition to an siRNA solution in ethanol‐containing citrate buffer (50 mM, pH 4.0). After incubation for 20 min, the mixture was sonicated, extruded through a 100 nm membrane, and dialyzed (30 nm pore) to remove free siRNA. The final product was lyophilized with a cryoprotectant (Figure 9A).

### 4.4 Physicochemical Characterization

#### 4.4.1 Size and Zeta Potential

Hydrodynamic diameter, polydispersity index (PDI), and zeta potential were measured in triplicate using dynamic light scattering (DLS, NanoBrook 90plus PALS, Brookhaven, USA).

#### 4.4.2 Transmission Electron Microscopy

Samples stained with 2% phosphotungstic acid were imaged by transmission electron microscopy (TEM, JEM‐1400plus, JEOL, Japan) at 80 kV.

#### 4.4.3 UV–Vis and Fluorescence Spectroscopy

Samples stained with 90 µL deionized water were analyzed by Multifunctional enzyme‐linked immunosorbent assay reader (Infinite E Plex, Tecan, Switzerland). Detection wavelength of UV–vis was 350–900 nm. Excitation wavelength of Fluorescence spectrum was 640 nm.

#### 4.4.4 Drug Loading and Encapsulation Efficiency

1% Triton X‐100 and LNP solution were mixed at room temperature. RNA concentration was measured using RediPlate 96 RiboGreen RNA quantification kit. The standard curve is shown in Figure S3B. Encapsulation efficiency (EE) and drug loading (DL) were calculated as:

$$\mathrm{EE}\left(\%\right)=\mathrm{siRNA}\;\mathrm{Mass}/\mathrm{Total}\;\mathrm{siRNA}\;\mathrm{Mass}\times 100$$

$$\mathrm{DL}\left(\%\right)=\mathrm{siRNA}\;\mathrm{Mass}/\mathrm{Total}\;\mathrm{Nanoparticle}\;\mathrm{Weight}\times 100$$

Results: EE ∼ siRNA ∼ = 93.1%5 ± 2.49%; DL∼siRNA∼ = 4.48% ± 0.11%.

#### 4.4.5 In Vitro Release Study of siRNA

The release kinetics of siRNA from the liposomes were evaluated using a nanodialysis system. Briefly, 2 mL of the sample was placed in the nanodialysis device and immersed in PBS release medium under room temperature. Aliquots were collected at 1, 2, 4, 8, 24, 48, and 72 h. The amount of released siRNA was quantified using the above RNA quantification kit, and the cumulative release percentage was calculated. The release curve was plotted as a function of time versus cumulative release rate.

### 4.5 Animal Models

Adult male C57BL/6 mice, aged 8 weeks (18–22 g), were acquired from Beijing Weitonglihua Experimental Animal Technology Co., Ltd. The mice were raised under standard conditions (25 ± 2°C, 12‐h light–dark cycle, free access to water and food). The animal experiments were approved by the Animal Care and Use Committees of the Laboratory Animal Research Center at China Medical University (approval number: KT20250379).

#### 4.5.1 Chronic Alcohol Exposure

After 1 week of acclimation, the mice were subjected to chronic alcohol exposure as previously described [10, 50, 54]. In brief, the mice received intragastric administration of water (control) or alcohol (5 g/kg, 25% ethanol w/v) daily for four consecutive weeks. Gavage needle with a bent end was used. Intragastric procedures were performed as gentle as possible to avoid choking of mice. The control mice went through the same intragastric procedures except that purified water was given instead of alcohol. The volume of pure water received by the control mice was the same as that of alcohol received by the experimental mice with the same weight. After treatment for 4 weeks, the mice were rested for 24 h and then subjected to behavioral tests or sacrificed (Figure 2A). In this study, we targeted the medial prefrontal cortex (mPFC), a region essential for higher‐order cognition and emotion. In rodent, the mPFC compromises the prelimbic (PL), infralimbic (IL), and anterior cingulate (ACC) cortices.

#### 4.5.2 siACSL1@LNP‐MR Treatment

The mice were divided into four groups as follows: (1) the sham group (0.9%NaCl, 5 g/kg/day, administered i.g., 4 weeks); (2) the siACSL1@LNP‐MR group (tail vein injection, 1 mg/kg/5 days, 4 weeks); (3) the EtOH group (25% ethanol w/v, 5 g/kg/day, administered i.g., 4 weeks); and (4) the EtOH + siACSL1@LNP‐MR group. siACSL1@LNP‐MR was diluted with a solvent of PBS. The mice in the sham group received administered i.g. with 0.9%NaCl of the same volume with the EtOH group. The mice in both the sham group and the EtOH group received tail vein injection of PBS (Figure 10A).

### 4.6 Behavior Analysis

#### 4.6.1 Y‐Maze

Spatial working memory was evaluated using a Y‐maze test. The apparatus consisted of three identical arms (30 × 8 × 15 cm) arranged at a 120°angle. Each mouse was allowed to move freely in the maze for 5 min. A spontaneous alternation was defined as three consecutive entries into three different arms. The exploration time and frequency in both the novel and familiar arms, as well as the number of spontaneous alternations, were recorded for each mouse. The maze was thoroughly cleaned with 75% alcohol between trials to eliminate olfactory cues.

#### 4.6.2 NOR

NOR test was conducted using a black opaque open‐field arena (50 × 50 × 40 cm) over a three‐period protocol comprising habituation, training, and testing phases. On the habituation period, mice were allowed to freely explore the empty arena for 2 h. During the training session, two identical objects (A and A) were placed in the arena, and mice were permitted to explore for 5 min and had a rest for 2 h. On the test period, one familiar object (A) was replaced with a novel object (B), which differed in size, shape, and color. The recognition index (RI) was calculated as follows: RI = [time exploring novel object B / (time exploring A + time exploring B)] × 100%. The arena and objects were cleaned with 75% alcohol between trials to remove olfactory cues.

#### 4.6.3 T‐Maze

The spatial working memory of mice was assessed using a T‐maze apparatus consisting of a start arm (50 × 12 × 20 cm) and two goal arms (40 × 12 × 20 cm). During the habituation phase, mice were allowed to freely explore the maze with both arms open and accessible to chocolate‐flavored food pellets placed at the end of each arm. In the training phase (5–7 days), each daily session comprised 10–15 trials consisting of a forced‐choice step where one arm was blocked and a pellet was placed in the open arm, followed by a free‐choice step where both arms were opened but only the previously blocked arm contained a pellet. A correct choice was recorded if the mouse entered the novel arm containing the reward. Incorrect choices led to confinement in the empty arm for 30 s. Each trial was separated by a 15‐s interval. During the test phase, each mouse performed 10 such forced‐free choice trials. The selection time and correct number of times were record. The percentage of correct choices was calculated as (number of correct entries / total trials) × 100%. The apparatus was cleaned with 75% ethanol after each mouse to remove olfactory cues.

#### 4.6.4 Radial Arm Maze

Spatial memory was evaluated using an eight‐arm radial maze. During the habituation phase, each mouse was allowed to freely explore the entire maze for 10 min with a food reward placed at the end of all eight arms. The training phase consisted of two sessions per day for 2–3 days, in which mice were trained to explore all arms and consume the rewards within 5 min per session. In the test phase, rewards were placed only in four fixed arms (e.g., arms 1, 2, 4, and 7). The number of arm entries, reward consumption, and the number of trials required to collect all four rewards were recorded. The test concluded when all four rewards had been consumed. The maze was cleaned with 75% ethanol after each trial to eliminate olfactory cues.

### 4.7 Untargeted Metabolism and Lipidomics

#### 4.7.1 Metabolomics Analysis

Metabolomic profiling was performed using liquid chromatography‐mass spectrometry (LC‐MS). Tissue samples were homogenized via bead beating and extracted with methanol, followed by fractional processing for both metabolomic and lipidomic analyses. Chromatographic separation was carried out in both positive and negative ion modes using a BEH C8 column and an HSS T3 column, respectively. The mobile phases consisted of water / acetonitrile with 0.1% formic acid for positive mode and water/methanol with 6.5 mM ammonium bicarbonate for negative mode, under gradient elution conditions. Full‐scan mass spectrometry was applied with spray voltages of 3.50 kV in positive ion mode and 3.00 kV in negative ion mode, at a mass resolution of 70,000. Data processing including peak identification and quantification was performed using Thermo Xcalibur and TraceFinder software. The relative standard deviation (RSD) of metabolites in quality control (QC) samples was below 30%, indicating good analytical stability.

#### 4.7.2 Lipidomics Analysis

Lipidomic analysis was also conducted using an LC‐MS platform equipped with a Vanquish UHPLC system coupled to a Q Exactive mass spectrometer. Tissue samples were homogenized by bead beating, extracted, and fractionated to obtain the supernatant for lipid analysis. Chromatographic separation was achieved on an ACQUITY UPLC C8 column using a gradient elution with mobile phases consisting of acetonitrile/water with 10 mM ammonium acetate and acetonitrile / isopropanol with 10 mM ammonium acetate, at a flow rate of 0.3 mL/min. Full MS^1^ scanning and data‐dependent MS^2^ acquisition were performed in both positive and negative ion modes, with spray voltages set at +3.5 and −3.0 kV, and mass scan ranges of m/z 180–2500 and m/z 120–1800, respectively. Data were processed using Xcalibur and TraceFinder software. An RSD of less than 30% for lipids in QC samples demonstrated satisfactory method reliability.

### 4.8 Cell Culture and Maintenance

The BV2 cell line is a widely used model system, derived from immortalized murine (mouse) microglia were cultured in Dulbecco's Modified Eagle Medium (DMEM; Gibco, C11995500BT) supplemented with 10% heat‐inactivated fetal bovine serum (FBS; Cell‐Box, CF‐01P‐02‐S), 100 IU/mL penicillin, and 100 µg/mL streptomycin (Gibco,15140‐122). Cells were maintained in a humidified incubator (37°C, 5% CO_2_) with medium replenishment every 48–72 h. All cell lines underwent routine mycoplasma screening (MycoAlert, Lonza) and were authenticated via short tandem repeat (STR) profiling prior to experimentation. Following established protocols [55, 56], chronic ethanol exposure was modeled by treating cells with 100 mM ethanol for 24 h.

### 4.9 Primary Microglia Cell Culture

Primary microglia were isolated and cultured as previously described [57]. Briefly, brains from P0‐2 newborn mouse pups were dissected and placed in ice‐cold dissection buffer (HBSS supplemented with 10 mM HEPES, 35 mM glucose, and 100 U/mL penicillin‐streptomycin). After careful removal o the meninges, cortical tissues were microdissected, transferred into 30 mL of fresh dissection buffer, and digested with 1.5 mL of trypsin for 15 min at 37°C. Digestion was halted by adding 1.2 mL of trypsin inhibitor (1 mg/mL) for 1 min, followed by 750 µL DNase (10 mg/mL) to reduce viscosity. The suspension was centrifuged at 400 g for 5 min. The pellet was gently triturated in 5 mL of microglia culture medium (DMEM containing 10% heat‐inactivated FBS and 100 U/mL penicillin‐streptomycin) and centrifuged again under identical conditions. After resuspension in 5 mL culture medium, cell density was determined using a hemocytometer. Cells were seeded into poly‐D‐lysine‐coated T‐75 flasks at a density of 5 × 10^4^ cells per mm^2^ (approximately 3–4 × 10^6^ cells per flask) and maintained at 37°C in a humidified incubator with 5% CO_2_. The medium was replaced 24 h after plating to remove non‐adherent debris and subsequently refreshed every 5 days. On day 10, microglia were harvested by vigorous tapping of the flasks, and detached cells in the supernatant were collected. The resulting cell population consisted of >95% microglia and was used for subsequent experiments.

### 4.10 Western Blot Analysis

Cells samples or mouse prefrontal cortex tissue samples were lysed using ice‐cold RIPA lysis buffer (Beyotime Institute of Biotechnology). The lysates were centrifuged to remove insoluble debris, and protein concentrations were determined with the Bradford assay (Bio‐Rad Laboratories). Equal amounts of protein were separated by SDS‐PAGE on 4%–12% Bis‐Tris NuPAGE gels (Invitrogen) at 200 V for 60 min. Subsequently, proteins were transferred onto nitrocellulose membranes and blocked with 3% bovine serum albumin (BSA) in TBST buffer (50 mM Tris, pH 8.0, 150 mM NaCl, 0.1% Tween‐20) for 1 h at room temperature. The membranes were incubated with primary antibodies against ACSL1 (1:1000, Invitrogen, PA5‐78713), IL‐1β (1:1000, Servicebio, GB111113), IL‐6 (1:1000, Servicebio, GB111117), NLRP3(1:1000, Servicebio, GB111113), PLIN2 (1:1000, Invitrogen, MA5‐32664), PTPRM (1:1000, Servicebio, GB114921) and β‐actin (1:1000, Ptoteintech, 66009‐1) overnight at 4°C. After three washes with TBST, membranes were incubated with horseradish peroxidase (HRP)‐conjugated secondary antibodies or streptavidin‐HRP for 1 h at room temperature. Following three additional washes with TBST, protein bands were visualized using an enhanced chemiluminescence (ECL) detection system.

### 4.11 qRT‐PCR

Total RNA was isolated using RNAiso Plus reagent (Takara). Then, 400 ng of RNA was reverse‐transcribed into cDNA using the PrimeScript II first Strand cDNA Synthesis Kit (Takara). Quantitative PCR (qPCR) was performed using SYBR Premix Ex Taq II (Takara) on a LightCycler 480 II Instrument (Roche). The primer sequences were as follows:

ACSL1‐Forward: 5’‐TGCCAGAGCTGATTGACATTC‐3’, Reverse: 5’‐GGCATACCAGAAGGTGGTGAG‐3’

Actin‐Forward: 5’‐GTGACGTTGACATCCGTAAAGA‐3’, Reverse: 5’‐GCCGGACTCATCGTACTCC‐3’.

The relative expression level of ACSL1 mRNA was calculated using the comparative ΔΔCt method, with GAPDH as the endogenous reference gene for normalization.

### 4.12 Chromogenic Immunohistochemistry, Fluorescent Immunohistochemistry, and Cell Quantifications Analysis

#### 4.12.1 Chromogenic Immunohistochemistry

Paraffin‐embedded mouse brain sections were deparaffinized, rehydrated, and subjected to antigen retrieval in EDTA buffer (pH 9.0) at 95°C for 35 min. After blocking with 3% BSA, sections were incubated with the following primary antibodies against NEUN (1:500, Abways, CY5515), IBA1 (1:2000, Abcam, ab178846), ACSL1 (1:500, Invitrogen, PA5‐78713), PLIN2 (1:500, Invitrogen, MA5‐32664), and PTPRM (1:500, Abcam, ab231607) at 4°C overnight, followed by HRP‐conjugated secondary antibody (Ruchuang, RCA054) and fluorescence development using a tyramide amplification kit (Ruchuang, RC0086‐12). Nuclei were stained with DAPI (Ruchuang, RC05), and slides were mounted with anti‐fade medium. Imaging was performed using a multi‐channel fluorescence scanner (KFbio, KF‐FL‐020) with channel‐specific wavelength settings. For quantification, representative images were acquired with KFSlide OS Analyzer software. All images for comparative analysis were acquired with identical laser power, gain, and exposure time settings.

#### 4.12.2 Fluorescent Immunohistochemistry

Following transcardial perfusion with ice‐cold PBS, brains were fixed in 4% formalin for 48 h, cryoprotected in 30% sucrose, and embedded in Tissue‐Tek O.C.T. compound. Coronal sections (30 µm) were prepared using a cryostat. Sections were blocked and permeabilized in PBS containing 5% donkey serum and 0.01% Triton X‐100. Primary antibodies were applied overnight at 4°C at the following dilution: anti‐IBA1 (Oasisbiofarm, OB‐PRB029‐02,1:400), anti‐ACSL1 (Invitrogen, PA5‐78713, 1:100), anti‐PLIN2 (Invitrogen, MA5‐32664, 1:100). The following day, sections were incubated with species‐matched secondary antibodies (Thermo Fisher) for 120 min at room temperature: Alexa Fluor 488 (1:200), Alexa Fluor 555 (1:200). Nuclei were counterstained with DAPI for 15 min at room temperature. Coverslips were mounted with using anti‐fade mounting medium (Solarbio, S2100). Images were acquired on a Leica TCS SP8 confocal microscope with a 63x oil‐immersion objective. Z‐stackswere collected at a step size of 0.5 µm to capture full cellular volumes. Image processing and minimal adjustments were performed using LAS X software (Leica) and Adobe Photoshop CS4.

#### 4.12.3 Cell Quantification and Contact Analysis

Cell densities for IBA1^+^, ACSL1^+^, PLIN2^+^, NEUN^+^, PTPRM^+^ cells were quantified manually according to established protocols [57, 58]. Microglia cell counts were normalized to the area of the region of interest (ROI) and expressed as cells per mm^2^. Analysis of cellular co‐localization was performed using Image J (version 2.9.0) as previously described [59]. For contact analysis between microglia and neurons, multichannel Z‐stack images were processed with Imaris software (version 9.9.0, Oxford Instruments). 3D surfaces were reconstructed for Iba1^+^ microglial processes and NeuN^+^ neuronal somata. A physical contact event was defined objectively when the shortest 3D distance between the surface of a microglial bulbous ending and the surface of a neuronal soma measured less than 1.0 µm. Each candidate contact was visually verified in three orthogonal planes (XY, XZ, YZ) within Imaris to confirm direct apposition, excluding false positives due to a tangential passage or signal noise. Contacts were manually counted by an investigator blinded to experimental conditions, with results recorded per neuronal soma. Neurons were subsequently categorized as contacted (colored in red) or uncontacted neurons (the number of contacts was 0, colored in pink). Data are expressed as the percentage of neurons exhibiting at least one microglial contact, in accordance with previously described approaches [57, 60].

### 4.13 Nile Red and BODIPY Staining

Cells were washed once with cold PBS and then fixed in 4% paraformaldehyde (PFA) for 20 min. The cells were washed three times with PBS, permeabilized with 0.25% Triton X‐100 for 15 min, and then washed three times. Thereafter, the slides were incubated with Nile Red (Solarbio, N8440) or BODIPY 493/503 (ThermoFisher, D3922) for 30 min. After three times washing with PBS, nuclei staining was then performed with DAPI (1:10000; AppliChem, Darmstadt, Germany). Finally, Vectashield (Vector Laboratories, H‐1000) was used for mounting.

### 4.14 Nanoparticle Treatment and Immunofluorescence Staining

Cells were seeded into confocal dishes and incubated for 24  h. After gentle washing with PBS, nanoparticles were diluted to 100  µg/mL in serum‐free medium, and 2  mL of the solution was added per dish. Following another 24  h incubation, the medium was aspirated and cells were fixed with 4% paraformaldehyde for 15  min at room temperature. After three PBS washes, cells were permeabilized with 0.1% Triton X‐100 for 30  min at room temperature and blocked with 5% donkey serum for 2  h at room temperature. Primary antibodies against IBA1 (Oasis biofarm, OB‐MMS039‐01) and NeuN (Proteintech, 66836‐1‐lg), each diluted 1:400, were applied and incubated overnight at 4°C. The following day, samples were equilibrated to room temperature for 30 min, washed three times with PBS, and incubated with Alexa Fluor 488‐conjugated secondary antibody (Invitrogen, A‐21202, 1:300) for 2 h at room temperature. After three final PBS washes, nuclei were counterstained with DAPI for 15 min at room temperature. Following three additional washes, samples were immediately imaged using a confocal microscope.

### 4.15 Statistical Analysis

Data are presented as mean ± SD. The animal sample size (n = 5–12 per group) was determined by statistical power analysis. For in vitro studies, *n* = 3 independent samples were used per group. Each data point represents an independent biological replicate.

All statistical analyses were performed using GraphPad Prism (version 10.0). Comparisons between two groups were conducted using a two‐tailed unpaired Student's t‐test. For comparisons among three or more groups, one‐way ANOVA followed by Tukey's post‐hoc test was employed. Statistical significance was defined at ^*^ *p* < 0.05, ^**^ *p* < 0.01, ^***^ *p*< 0.001, ^****^ *p*< 0.0001; n.s. indicates non‐significant results. The specific statistical test applied in each experiment is detailed in the respective figure legend. Complete statistical outputs, including exact test values (t‐value with degrees of freedom [df], or F‐value with both degrees of freedom) and P‐values, are provided in Supporting Information.

The acquired metabolomics and lipidomics data were processed using Thermo Qual Browser (Xcalibur 2.2 SP1.48) for qualitative identification and retention time confirmation. A peak table was generated and imported into TraceFinder 5.1 software. A quantitative method was established based on the built‐in Compound Database to perform targeted quantification.

## Author Contributions

L.H. and H.W. conceived the study. B.L. and R.G. performed the bioinformatics analysis on the GEO dataset. L.H. and F.Z. drafted the manuscript. L.H., X.C. and J.W. carried out all animal experiments and related data analysis. Z.C. supervised the entire research. Z.D. and H.W. provided funding, and critically revised the manuscript for important intellectual content. All authors read and approved the final manuscript.

## Funding

This research was partly funded by National Natural Science Foundation of China (Grant No. 82373013) and Natural Science Foundation of Liaoning Province (No. 2024JH6/100800018).

## Ethics Statement

Not applicable for the human data component, as it involved analysis of public dataset GSE141552. All animal procedures were approved by the Institutional Animal Care and Use Committee (IACUC) of China Medical University (Approval Number: 20250379) and performed in accordance with the NIH Guide for the Care and Use of Laboratory Animals.

## Consent

We confirm that this manuscript is the authors’ original work and has not been published nor has it been submitted simultaneously elsewhere. All authors have read and approved the final version of the manuscript.

## Conflicts of Interest

The authors declare no conflicts of interest.

## Supporting information




**Supporting File 1**: advs74251‐sup‐0001‐SuppMat.docx.

**Supporting File 2**: advs74251‐sup‐0002‐Table.docx.

## Data Availability

The human dataset (GSE141552) analyzed during the current study is available in the Gene Expression Omnibus (GEO) repository. The data generated from the animal experiments in this study are available from the corresponding author on reasonable request.
