# Supplementary material for: ACSL1‐Dependent Microglial Lipoimmunometabolic Reprogramming Underlies Cognitive Deficits in Alcohol Use Disorder
Source: Adv Sci (Weinh). 2026 Feb 5;13(21):e19760. doi: 10.1002/advs.202519760 (PMC13073305; doi:10.1002/advs.202519760)
Supplement: Supplementary file 1 — Supporting File 1: advs74251‐sup‐0001‐SuppMat.docx. [file ADVS-13-e19760-s001.docx]

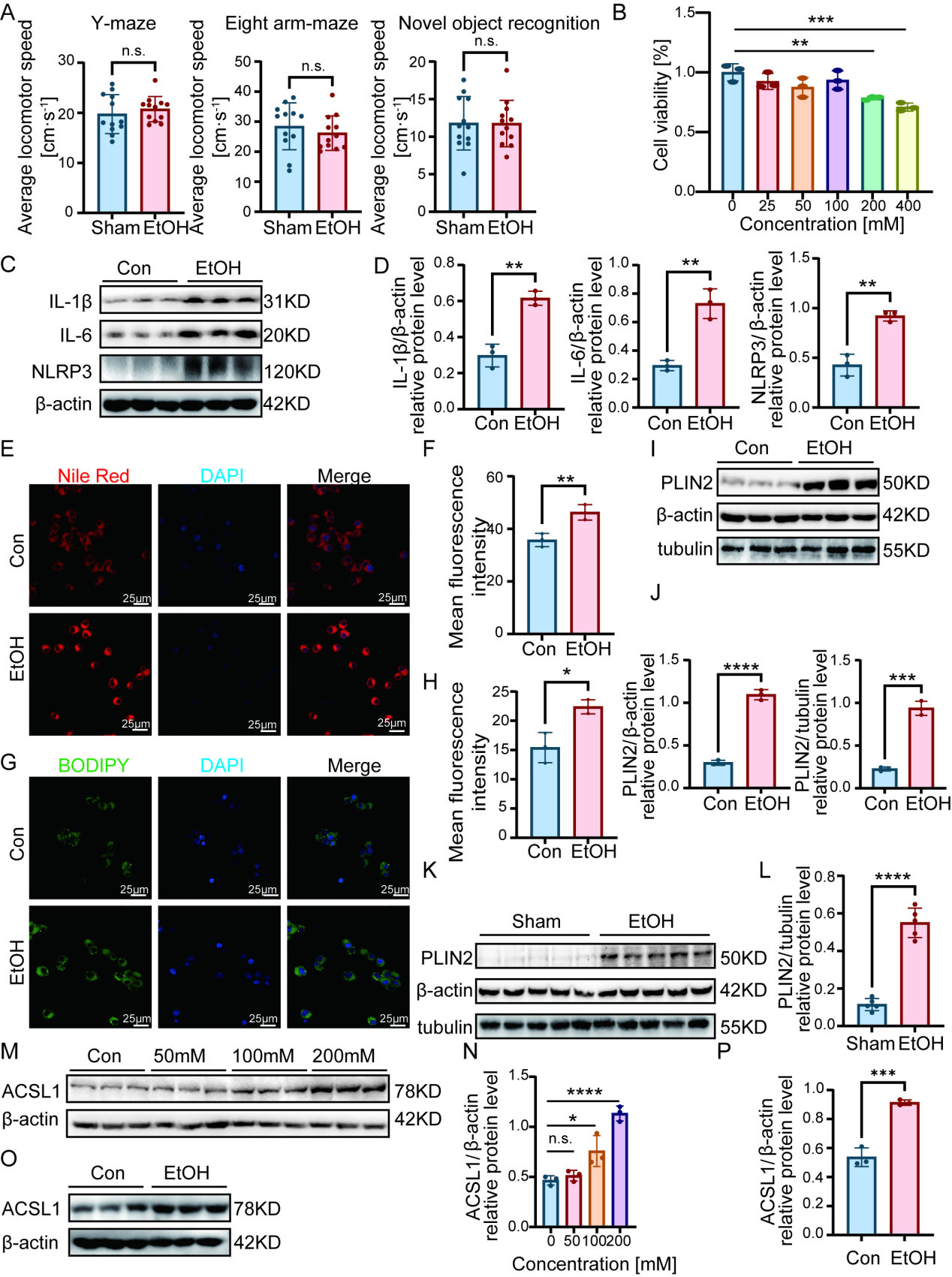


Supplementary Figure 1

(**A**) Average locomotor speed of ethanol-exposed and control mice in Behavioral cognitive assessments of Y-maze, 8-arm radial maze and novel object recognition. n = 12 mice per group. **(B)** CCK-8 assay of BV2 cells treated with different concentration ethanol. **(C-D)** Western blot analysis confirms upregulation of IL-1β, IL-6 and NLRP3 in ethanol-treated BV2 cells. **(E-H)** Representative images and quantification of neutral lipid (Nile Red) and total lipid (BODIPY) staining in control and ethanol-treated BV2 cells. 5 randomly selected fields per independent experiment. **(I-L)** Western blot analysis confirming significant upregulation of PLIN2 protein in ethanol-treated BV2 cells and mice model, β-actin and tubulin as internal reference proteins respectively. **(M-N)** Western blot analysis of ACSL1 protein in control and different concentration ethanol-treated BV2 cells. **(O-P)** Western blot analysis confirms ACSL1 protein is upregulated in ethanol-treated BV2 cells. For in-vivo studies **(K-N)**: n = 5 mice per group. For in-vitro studies **(B-L&O-P)**: n = 3 independent samples per group. All bar graphs data are presented as mean ± SD.**P*< 0.05, ***P*< 0.01, ****P*< 0.001, *****P*< 0.0001, n.s. nonsignificant; by two-tailed unpaired t-test. Scale bars, as shown in the figure.


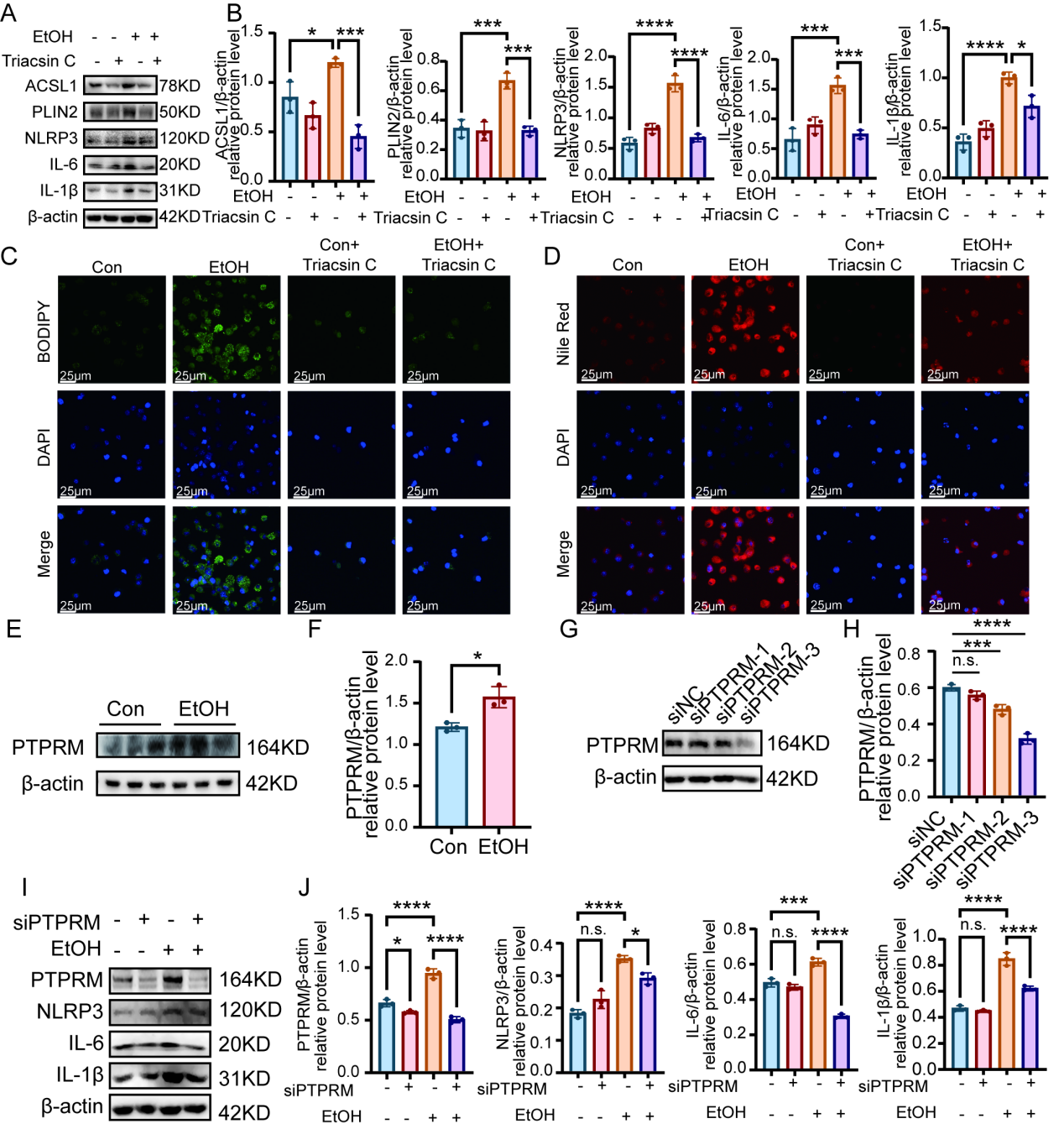


Supplementary Figure 2

**(A-B)** Western blot analysis shows Triacsin C treatment reverses ethanol-induced upregulation of ACSL1, inflammatory markers (NLRP3, IL-1β, IL-6), and PLIN2 in BV2 cells. **(C-D)** Quantification of Nile Red (neutral lipid) and BODIPY (total lipid) fluorescence shows Triacsin C reduces ethanol-induced lipid accumulation in BV2 microglia cells. 5 randomly selected fields per independent experiment. **(E-F)** Western blot analysis confirms PTPRM protein is upregulated in ethanol-treated BV2 cells. **(G-H)** Construction of PTPRM knockdown primary microglia with siRNA and validation by Western blot. **(I-J)** Western blot analysis shows PTPRM knockdown reverses ethanol-induced upregulation of inflammatory markers PTPRM, NLRP3, IL-1β and IL-6 in BV2 cells. Data are presented as mean ± SD. For in-vitro studies: n = 3 independent experiments. **P*< 0.05, ****P*< 0.001, *****P*< 0.0001, n.s. nonsignificant; **(F&H)** by two-tailed unpaired t-test. **(B&J)** by one-way ANOVA with Tukey's post hoc test. Scale bars, as shown in the figure.


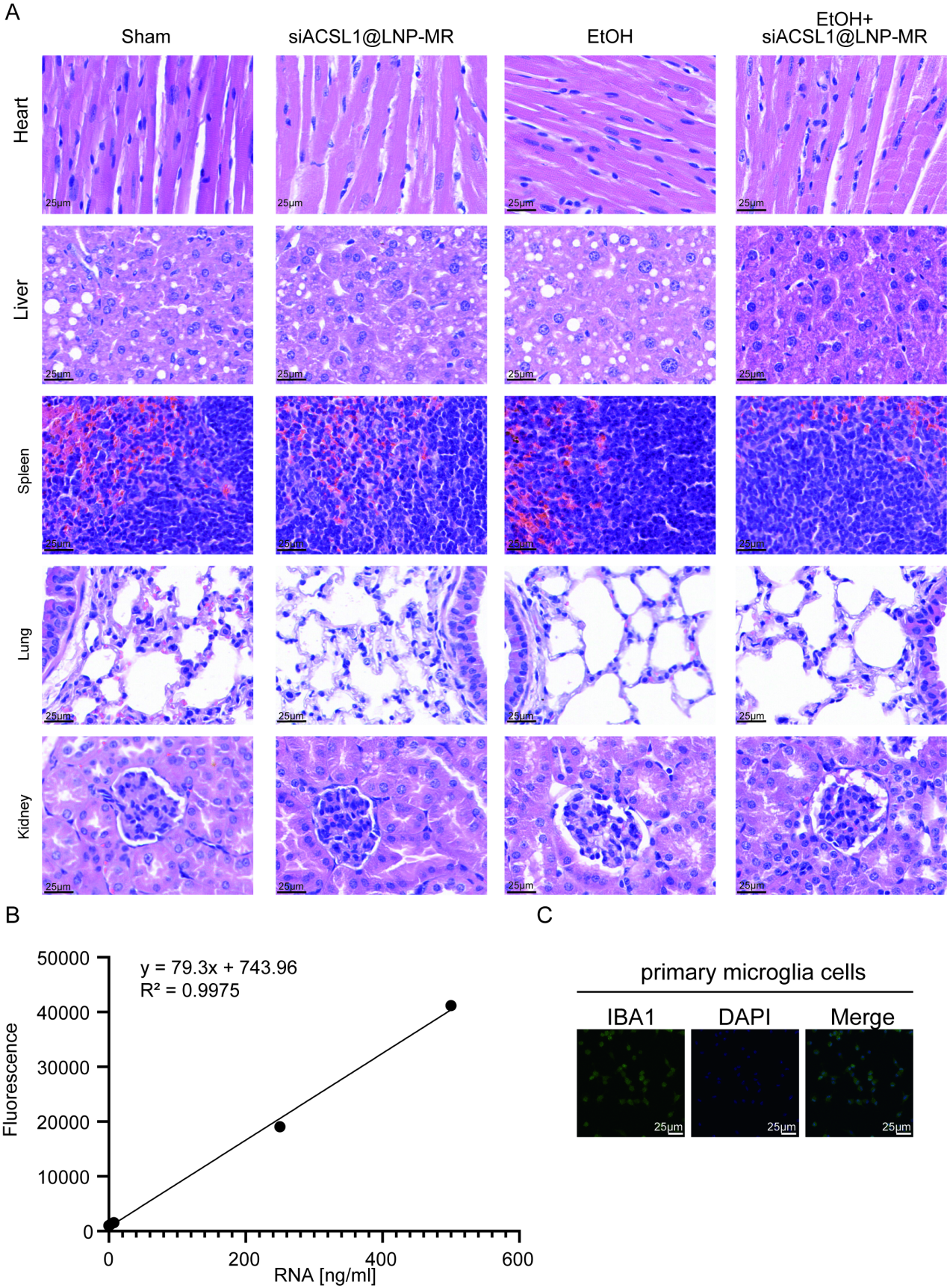


Supplementary Figure 3

**(A)** Representative the picture of HE staining of heart, liver, spleen, lungs and kidneys treated with siACSL1@LNP-MR or other groups. **(B)** Standard curve validation for RNA quantification. **(C)** Representative image of IBA1 immunofluorescence of primary microglia. Scale bars, as shown in the figure.
