# Supplementary material for: ACSL1‐Dependent Microglial Lipoimmunometabolic Reprogramming Underlies Cognitive Deficits in Alcohol Use Disorder
Source: Adv Sci (Weinh). 2026 Feb 5;13(21):e19760. doi: 10.1002/advs.202519760 (PMC13073305; doi:10.1002/advs.202519760)
Supplement: Supplementary file 2 — Supporting File 2: advs74251‐sup‐0002‐Table.docx. [file ADVS-13-e19760-s002.docx]

| **Table of Data Statistics Information** | | | | | | | |
| --- | --- | --- | --- | --- | --- | --- | --- |
| Figure Number | Sample size (n) | Mean ± SD | Statistical Test | Variable: Degrees of Freedom | Comparative Description | *P* value | Significance Marking |
| Figure 2B | n=12 mice per group | Time:  Sham: 3.308±0.8051  EtOH: 9.900±2.552 | two-tailed unpaired t-test | Time: t (22) =8.534 | Sham vs. EtOH | <0.0001 | **** |
|  |  | Accuracy:  Sham: 71.67±12.67  EtOH: 61.67±9.374 |  | Accuracy: t (22) =2.198 |  | 0.0388 | * |
| Figure 2D | n=12 mice per group | Entries:  Sham: 48.56±5.647  EtOH: 42.97±5.224 | two-tailed unpaired t-test | Entries: t (22) =2.521 | Sham vs. EtOH | 0.0195 | * |
|  |  | Time:  Sham: 51.06±11.60  EtOH: 27.73±12.00 |  | Time: t (22) =4.841 |  | <0.0001 | **** |
| Figure 2F | n=12 mice per group | Working memory errors:  Sham: 15.09±5.818  EtOH: 35.98±17.932 | two-tailed unpaired t-test | Working memory errors: t (22) =7.353 | Sham vs. EtOH | <0.0001 | **** |
|  |  | Reference memory errors:  Sham: 15.41±6.055  EtOH: 32.30±7.838 |  | Reference memory errors: t (22) =5.909 |  | <0.0001 | **** |
| Figure 2H | n=12 mice per group | Sham: 57.43±14.52  EtOH: 41.13±17.73 | two-tailed unpaired t-test | Recognition index: t (22) =2.464 | Sham vs. EtOH | 0.0220 | * |
| Figure 3B | n=5 mice per group | Sham: 0.5105±0.2146  EtOH: 0.9355±0.1860 | two-tailed unpaired t-test | IL-1β: t (8) =3.933 | Sham vs. EtOH | 0.0043 | ** |
|  |  | Sham: 0.2470±0.1220  EtOH: 0.4690±0.09612 |  | IL-6: t (8) =3.196 |  | 0.0127 | * |
|  |  | Sham: 0.3355±0.1081  EtOH: 0.9041±0.2036 |  | NLRP3: t (8) =5.515 |  | 0.0006 | *** |
| Figure 3D | n=5 mice per group | Sham: 35.77±2.781  EtOH: 59.07±2.555 | two-tailed unpaired t-test | IBA1+ Percentage:  t (8) =13.97 | Sham vs. EtOH | <0.0001 | **** |
|  |  | Sham: 21.04±2.425  EtOH: 36.68±3.980 |  | Soma diameter:  t (8) =7.504 | Sham vs. EtOH | <0.0001 | **** |
| Figure 3F | n=3 independent samples per group | Con: 0.8945±0.03485  EtOH: 1.186±0.1272 | two-tailed unpaired t-test | IL-1β: t (4) =3.830 | Con vs. EtOH | 0.0186 | * |
|  |  | Con: 0.08521±0.01258  EtOH: 0.8776±0.08822 |  | IL-6: t (4) =15.40 |  | 0.0001 | *** |
|  |  | Con: 0.5001±0.1299  EtOH: 0.7482±0.1133 |  | NLRP3: t (4) =2.494 |  | 0.0672 | n.s. |
| Figure 4C | n=6 mice per group | Sham: 0.2169±0.06422  EtOH: 0.3139±0.06165 | two-tailed unpaired t-test | Palmitoleic acid:  t (10) =2.669 | Sham vs. EtOH | 0.0235 | * |
|  |  | Sham: 0.05771±0.01155  EtOH: 0.04286±0.007617 |  | Pantadecatrienoic acid: t (10) =2.630 |  | 0.0252 | * |
| Figure 4G | n=6 mice per group | Sham: 60369±11062  EtOH: 72865±4664 | two-tailed unpaired t-test | FA: t (10) =2.550 | Sham vs. EtOH | 0.0289 | * |
|  |  | Sham: 666.9±162.7  EtOH: 888.2±61.27 |  | OAHFA: t (10) =3.119 |  | 0.0109 | * |
|  |  | Sham: 113853±19709  EtOH: 132213±11729 |  | PC: t (10) =1.961 |  | 0.0783 | n.s. |
|  |  | Sham: 2547±273.8  EtOH: 3109±167.6 |  | PG: t (10) =4.290 |  | 0.0016 | ** |
|  |  | Sham: 853.6±134.8  EtOH: 838.2±144.5 |  | TG: t (10) =0.1913 |  | 0.8521 | n.s. |
|  |  | Sham: 55893±6029  EtOH: 60642±4289 |  | PE: t (10) =1.572 |  | 0.1470 | n.s. |
| Figure 5C | n=5 mice per group | Sham: 4.631±1.317  EtOH: 24.16±1.703 | two-tailed unpaired t-test | t (4) =15.71 | Sham vs. EtOH | <0.0001 | **** |
| Figure 5E | n=3 independent samples per group | Con: 13.23±1.858  EtOH: 69.00±2.839 | two-tailed unpaired t-test | t (4) =28.47 | Con vs. EtOH | <0.0001 | **** |
| Figure 5G | n=3 independent samples per group | Con: 43.38±3.373  EtOH: 61.55±2.090 | two-tailed unpaired t-test | t (4) =7.931 | Con vs. EtOH | 0.0014 | ** |
| Figure 5I | n=5 mice per group | Sham: 0.1328±0.06573  EtOH: 0.6486±0.9700 | two-tailed unpaired t-test | t (8) =9.843 | Sham vs. EtOH | <0.0001 | **** |
| Figure 5K | n=3 independent samples per group | Con: 0.3582±0.1386  EtOH: 1.361±0.06250 | two-tailed unpaired t-test | t (4) =11.42 | Con vs. EtOH | 0.0003 | *** |
| Figure 6E | n=5 mice per group | Sham: 0.5212±0.05291  EtOH: 0.9113±0.08241 | two-tailed unpaired t-test | t (8) =8.908 | Sham vs. EtOH | <0.0001 | **** |
| Figure 6G | n=3 independent samples per group | Con: 0.2400±0.08754  EtOH: 0.8904±0.1379 | two-tailed unpaired t-test | t (4) =6.898 | Con vs. EtOH | 0.0023 | ** |
| Figure 6I | n=5 mice per group | Con: 20.71±1.454  EtOH: 29.02±1.730 | two-tailed unpaired t-test | t (8) =8.216 | Sham vs. EtOH | <0.0001 | **** |
| Figure 7E | n=3 independent samples per group | From left to right  Group1: 0.4804±0.01891  Group2: 0.4523±0.02354  Group3: 0.7090±0.01823  Group4: 0.5929±0.01121 | one-way ANOVA with Tukey's post hoc test | ACSL1: F (3,8) =120.4 | Group1 vs. Group3 | <0.0001 | **** |
|  |  |  |  |  | Group3 vs. Group4 | 0.0003 | *** |
|  |  | From left to right  Group1: 0.9736±0.02562  Group2: 0.8300±0.05573  Group3: 1.117±0.06125  Group4: 0.9378±0.01575 |  | PLIN2: F (3,8) =21.74 | Group1 vs. Group3 | 0.0169 | * |
|  |  |  |  |  | Group3 vs. Group4 | 0.0047 | ** |
|  |  | From left to right  Group1: 0.2163±0.01582  Group2: 0.2194±0.02537  Group3: 0.7356±0.01260  Group4: 0.6443±0.01679 |  | NLRP3: F (3,8) =680.3 | Group1 vs. Group3 | <0.0001 | **** |
|  |  |  |  |  | Group3 vs. Group4 | 0.0013 | ** |
|  |  | From left to right  Group1: 0.3483±0.03014  Group2: 0.3783±0.02021  Group3: 0.6777±0.02323  Group4: 0.5148±0.02416 |  | IL-6: F (3,8) =111.4 | Group1 vs. Group3 | <0.0001 | **** |
|  |  |  |  |  | Group3 vs. Group4 | 0.0002 | *** |
|  |  | From left to right  Group1: 0.3169±0.01535  Group2: 0.3283±0.01758  Group3: 0.6449±0.02301  Group4: 0.2802±0.01264 |  | IL-1β: F (3,8) =279.3 | Group1 vs. Group3 | <0.0001 | **** |
|  |  |  |  |  | Group3 vs. Group4 | <0.0001 | **** |
| Figure 8E | n=3 independent samples per group | Con: 0.5215±0.06457  EtOH: 1.110±0.06954 | two-tailed unpaired t-test | t (4) = 10.73 | Con vs. EtOH | 0.0004 | *** |
| Figure 8G | n=5 mice per group | Sham: 0.4918±0.1306  EtOH: 0.7890±0.09210 | two-tailed unpaired t-test | t (8) = 4.159 | Sham vs. EtOH | 0.0032 | ** |
| Figure 8K | n=5 mice per group | Sham: 32.50±2.464  EtOH: 45.50±3.736 | two-tailed unpaired t-test | t (8) =6.499 | Sham vs. EtOH | 0.0002 | *** |
| Figure 8M | n=5 mice per group | Sham: 26.46±3.172  EtOH: 50.60±2.364 | two-tailed unpaired t-test | t (8) = 13.64 | Sham vs. EtOH | <0.0001 | **** |
| Figure 9C | n=3 independent samples per group | LNP: 41.39±2.547  LNP-MR: 36.87±2.465 |  |  |  |  |  |
| Figure 10D | n=10 mice per group | From left to right  Group1: 35.28±1.756  Group2: 36.14±4.561  Group3: 33.07±3.063  Group4: 37.43±3.785 | one-way ANOVA with Tukey's post hoc test | Novel Entries/Total:  F (3,36) =2.828 | Group1 vs. Group2 | 0.9440 | n.s. |
|  |  |  |  |  | Group1 vs. Group3 | 0.4858 | n.s. |
|  |  |  |  |  | Group3 vs. Group4 | 0.0366 | * |
|  |  | From left to right  Group1: 27.14±2.994  Group2: 22.72±2.305  Group3: 9.690±3.742  Group4: 23.05±6.221 | one-way ANOVA with Tukey's post hoc test | Time in Novel/Total:  F (3,36) =34.30) | Group1 vs. Group2 | 0.0922 | n.s. |
|  |  |  |  |  | Group1 vs. Group3 | <0.0001 | **** |
|  |  |  |  |  | Group3 vs. Group4 | <0.0001 | **** |
| Figure 10F | n=10 mice per group | From left to right  Group1: 62.73±8.277  Group2: 59.17±5.626  Group3: 51.88±5.202  Group4: 63.85±6.569 | one-way ANOVA with Tukey's post hoc test | F (3,36) =6.845 | Group1 vs. Group2 | 0.6204 | n.s. |
|  |  |  |  |  | Group1 vs. Group3 | 0.0037 | ** |
|  |  |  |  |  | Group3 vs. Group4 | 0.0012 | ** |
| Figure 10H | n=10 mice per group | From left to right  Group1: 25.39±5.696  Group2: 24.87±4.116  Group3: 33.78±4.199  Group4: 27.39±4.289 | one-way ANOVA with Tukey's post hoc test | Working memory errors:  F (3,36) =7.852 | Group1 vs. Group2 | 0.9944 | n.s. |
|  |  |  |  |  | Group1 vs. Group3 | 0.0014 | ** |
|  |  |  |  |  | Group3 vs. Group4 | 0.0191 | * |
|  |  | From left to right  Group1: 15.40±6.155  Group2: 16.86±5.898  Group3: 36.70±2.703  Group4: 18.83±5.690 |  | Reference memory errors: F (3,36) =35.14 | Group1 vs. Group2 | 0.9264 | n.s. |
|  |  |  |  |  | Group1 vs. Group3 | <0.0001 | **** |
|  |  |  |  |  | Group3 vs. Group4 | <0.0001 | **** |
| Figure 11B | n=5 mice per group | From left to right  Group1: 29.28±1.050  Group2: 29.87±0.9414  Group3: 49.40±2.217  Group4: 35.76±0.7798 | one-way ANOVA with Tukey's post hoc test | IBA+%:  F (3,16) =175.7 | Group1 vs. Group2 | 0.9334 | n.s. |
|  |  |  |  |  | Group1 vs. Group3 | <0.0001 | **** |
|  |  |  |  |  | Group3 vs. Group4 | <0.0001 | **** |
|  |  | From left to right  Group1: 22.73±1.744  Group2: 25.00±2.799  Group3: 37.38±1.399  Group4: 24.91±1.646 |  | Diameter:  F (3,16) =57.77 | Group1 vs. Group2 | 0.2945 | n.s. |
|  |  |  |  |  | Group1 vs. Group3 | <0.0001 | **** |
|  |  |  |  |  | Group3 vs. Group4 | <0.0001 | **** |
| Figure 11C | n=5 mice per group | From left to right  Group1: 11.31±1.386  Group2: 9.020±1.023  Group3: 16.77±0.7617  Group4: 9.906±0.4991 | one-way ANOVA with Tukey's post hoc test | F (3,16) =63.59 | Group1 vs. Group2 | 0.0091 | ** |
|  |  |  |  |  | Group1 vs. Group3 | <0.0001 | **** |
|  |  |  |  |  | Group3 vs. Group4 | <0.0001 | **** |
| Figure 11E | n=5 mice per group | From left to right  Group1: 5.969±1.795  Group2: 5.274±1.433  Group3: 26.62±2.559  Group4: 6.358±1.726 | one-way ANOVA with Tukey's post hoc test | F (3,16) =145.7 | Group1 vs. Group2 | 0.9392 | n.s. |
|  |  |  |  |  | Group1 vs. Group3 | <0.0001 | **** |
|  |  |  |  |  | Group3 vs. Group4 | <0.0001 | **** |
| Figure 11G | n=5 mice per group | From left to right  Group1: 29.34±0.945  Group2: 22.20±1.056  Group3: 54.92±5.122  Group4: 16.62±0.8927 | one-way ANOVA with Tukey's post hoc test | Soma-soma interaction%: F (3,16) =197.2 | Group1 vs. Group2 | 0.0035 | ** |
|  |  |  |  |  | Group1 vs. Group3 | <0.0001 | **** |
|  |  |  |  |  | Group3 vs. Group4 | <0.0001 | **** |
|  |  | From left to right  Group1: 20.52±1.758  Group2: 17.28±1.479  Group3: 23.56±1.606  Group4: 18.84±0.8629 | one-way ANOVA with Tukey's post hoc test | PTPRM: F (3,16) =16.82 | Group1 vs. Group2 | 0.0144 | * |
|  |  |  |  |  | Group1 vs. Group3 | 0.0217 | * |
|  |  |  |  |  | Group3 vs. Group4 | 0.0006 | *** |
